# Climate Teleconnections and Recent Patterns of Human and Animal Disease Outbreaks

**DOI:** 10.1371/journal.pntd.0001465

**Published:** 2012-01-24

**Authors:** Assaf Anyamba, Kenneth J. Linthicum, Jennifer L. Small, Kathrine M. Collins, Compton J. Tucker, Edwin W. Pak, Seth C. Britch, James Ronald Eastman, Jorge E. Pinzon, Kevin L. Russell

**Affiliations:** 1 Biospheric Sciences Laboratory, NASA Goddard Space Flight Center, Greenbelt, Maryland, United States of America; 2 USDA-ARS Center for Medical, Agricultural, & Veterinary Entomology, Gainesville, Florida, United States of America; 3 Clark Labs, Clark University, Worcester, Massachusetts, United States of America; 4 Division of GEIS Operations, Armed Forces Health Surveillance Center, Silver Spring, Maryland, United States of America; National Institute of Parasitic Diseases China CDC, China

## Abstract

**Background:**

Recent clusters of outbreaks of mosquito-borne diseases (Rift Valley fever and chikungunya) in Africa and parts of the Indian Ocean islands illustrate how interannual climate variability influences the changing risk patterns of disease outbreaks. Although Rift Valley fever outbreaks have been known to follow periods of above-normal rainfall, the timing of the outbreak events has largely been unknown. Similarly, there is inadequate knowledge on climate drivers of chikungunya outbreaks. We analyze a variety of climate and satellite-derived vegetation measurements to explain the coupling between patterns of climate variability and disease outbreaks of Rift Valley fever and chikungunya.

**Methods and Findings:**

We derived a teleconnections map by correlating long-term monthly global precipitation data with the NINO3.4 sea surface temperature (SST) anomaly index. This map identifies regional hot-spots where rainfall variability may have an influence on the ecology of vector borne disease. Among the regions are Eastern and Southern Africa where outbreaks of chikungunya and Rift Valley fever occurred 2004–2009. Chikungunya and Rift Valley fever case locations were mapped to corresponding climate data anomalies to understand associations between specific anomaly patterns in ecological and climate variables and disease outbreak patterns through space and time. From these maps we explored associations among Rift Valley fever disease occurrence locations and cumulative rainfall and vegetation index anomalies. We illustrated the time lag between the driving climate conditions and the timing of the first case of Rift Valley fever. Results showed that reported outbreaks of Rift Valley fever occurred after ∼3–4 months of sustained above-normal rainfall and associated green-up in vegetation, conditions ideal for Rift Valley fever mosquito vectors. For chikungunya we explored associations among surface air temperature, precipitation anomalies, and chikungunya outbreak locations. We found that chikungunya outbreaks occurred under conditions of anomalously high temperatures and drought over Eastern Africa. However, in Southeast Asia, chikungunya outbreaks were negatively correlated (*p*<0.05) with drought conditions, but positively correlated with warmer-than-normal temperatures and rainfall.

**Conclusions/Significance:**

Extremes in climate conditions forced by the *El Niño*/Southern Oscillation (ENSO) lead to severe droughts or floods, ideal ecological conditions for disease vectors to emerge, and may result in epizootics and epidemics of Rift Valley fever and chikungunya. However, the immune status of livestock (Rift Valley fever) and human (chikungunya) populations is a factor that is largely unknown but very likely plays a role in the spatial-temporal patterns of these disease outbreaks. As the frequency and severity of extremes in climate increase, the potential for globalization of vectors and disease is likely to accelerate. Understanding the underlying patterns of global and regional climate variability and their impacts on ecological drivers of vector-borne diseases is critical in long-range planning of appropriate disease and disease-vector response, control, and mitigation strategies.

## Author Summary

Interannual climate variability associated with the *El Niño*/Southern Oscillation (ENSO) phenomenon and regional climatic circulation mechanisms in the equatorial Indian Ocean result in significant rainfall and ecological anomaly patterns that are major drivers of spatial and temporal patterns of mosquito-borne disease outbreaks. Correlation and regression analyses of long time series rainfall, vegetation index, and temperature data show that large scale anomalies occur periodically that may influence mosquito vector populations and thus spatial and temporal patterns of Rift Valley fever and chikungunya outbreaks. Rift Valley fever outbreak events occurred after a period of ∼3–4 months of persistent and above-normal rainfall that enabled vector habitats to flourish. On the other hand, chikungunya outbreaks occurred during periods of high temperatures and severe drought over East Africa and the western Indian Ocean islands. This is consistent with highly populated environmental settings where domestic and peri-domestic stored water containers were the likely mosquito sources. However, in Southeast Asia, approximately 52% of chikungunya outbreaks occurred during cooler-than-normal temperatures and were significantly negatively correlated with drought. Besides climate variability, other factors not accounted for such as vertebrate host immunity may contribute to spatio-temporal patterns of outbreaks.

## Introduction

Climate fluctuations leading to extreme temperatures, storm surges, flooding, and droughts produce conditions that precipitate mosquito-borne disease epidemics directly affecting global public health. Abnormally high temperatures affect populations of mosquito disease vectors by influencing: mosquito survival; susceptibility of mosquitoes to viruses; mosquito population growth rate, distribution, and seasonality; replication and extrinsic incubation period of a virus in the mosquito; and virus transmission patterns and seasonality [1], [2]. Extreme increases in precipitation may increase mosquito larval habitats or create new habitats and an overall increase in mosquito vector populations. For instance, the probability of vector survival can increase with humidity [1]–[5]. Unusually low rainfall or drought can also change habitats by concentrating water into small pools, potentially increasing the proportion of container breeding mosquito vectors. Concurrently, these anomalous patterns of temperature and precipitation have impacts on the vertebrate hosts of disease vector mosquitoes. Increased rain can increase vegetation, habitat, food availability, and thus survival of vertebrate host populations. Decreased rain can severely reduce or eliminate food resources forcing vectors and vertebrate hosts into human settlements, increasing vector-human contact [1]–[7].

**Figure 1:**
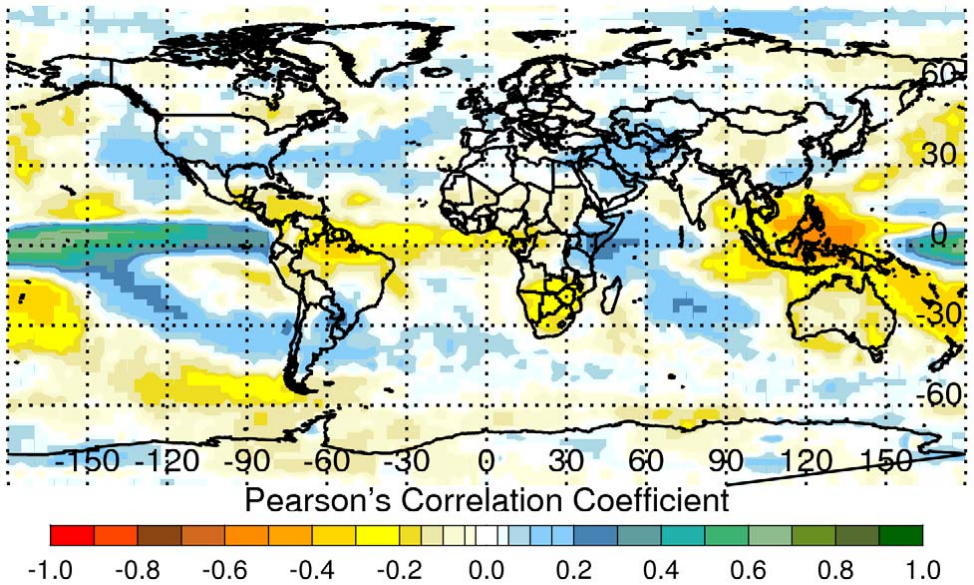
Summary correlation map between monthly NINO3.4 SST and rainfall anomalies, 1979–2008. Correlation of sea surface temperatures and rainfall anomalies illustrate ENSO teleconnection patterns. There is a tendency for above (below) normal rainfall during *El Niño* (*La Niña*) events over East Africa (Southern Africa, Southeast Asia). Similar differential anomaly patterns were observed for other regions, especially within the global tropics. These extremes (above or below) in rainfall influence regional ecology and consequently dynamics of mosquito disease vector populations and patterns of mosquito-borne disease outbreaks.

The *El Niño*/Southern Oscillation (ENSO) phenomenon is a well known climate fluctuation that is associated with extremes in the global climate regime. Over the last 30 years, a number of studies have shown that climate variability associated with the ENSO phenomenon influences several human and animal disease outbreaks including Rift Valley fever, Murray Valley encephalitis, chikungunya, malaria, Hantavirus pulmonary syndrome, and various other diseases [8]–[14]. ENSO is part of the earth's climate mechanism as inferred from various reconstructions of millions of years of climate proxy data [15]. The influence of ENSO on the global climate system, especially over the global tropics, through interannual variations in temperature, atmospheric circulation, and precipitation at various distant locations is termed *teleconnection*. Teleconnections produce differential anomaly patterns in these major climate variables with the near-cyclical transitions through time from the warm *El Niño* phase to the cold *La Niña* phase at the regional level around the world (Fig. 1). Teleconnections result from the coupling between oceanic and atmospheric components of the earth's climate system.

Periodic anomalous warming and cooling in the tropical central to eastern Pacific Ocean region (ENSO) can trigger a tropospheric bridge effect that propagates globally. This propagation is what triggers teleconnections and is postulated to have a lagged response of between 3–5 months. The effect of these teleconnections is that they can trigger anomalous convective activity, or lack of, at huge distances from the original site of warming [15]–[17]. These effects can be and are indeed modulated by coupled regional circulation patterns [18], [19]. The tendency for clusters of mosquito-borne diseases to occur simultaneously or a few months apart during an ENSO cycle shows how extremes in rainfall resulting in either persistent flood or severe drought can influence regional ecology and consequently dynamics of mosquito vector populations at distant locations around the world, especially in the tropics [10], [11], [13], [20].

**Figure 2:**
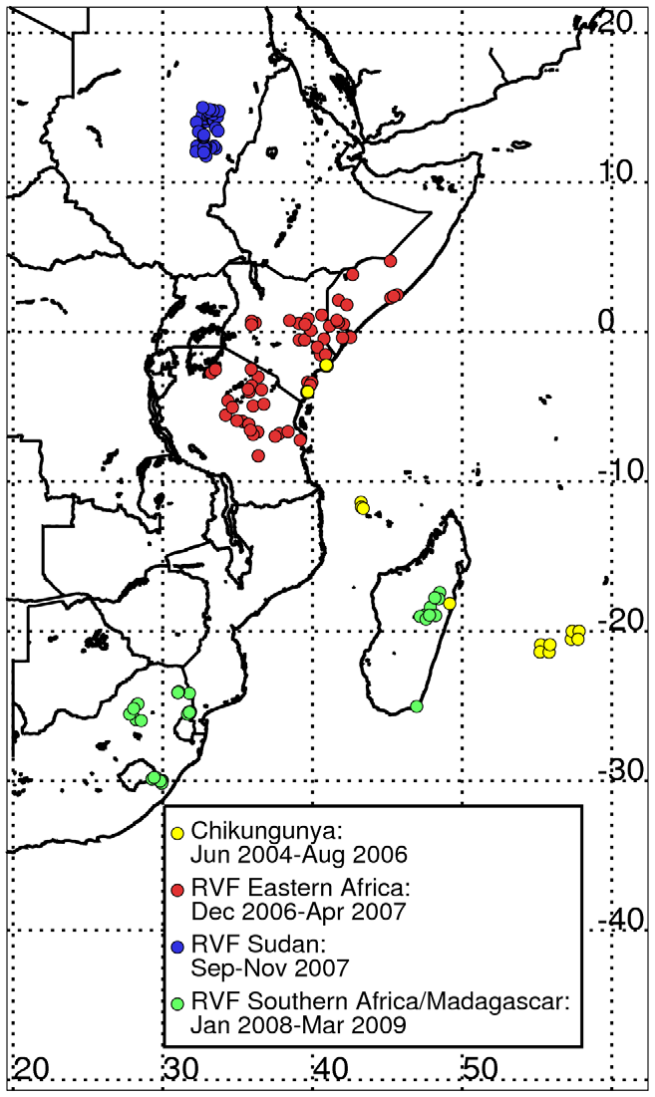
Outbreak locations of chikungunya (2004–2006) and Rift Valley fever (2006–2009). Symbols indicate distribution of recent outbreaks of chikungunya (2004–2006) shown by yellow dots and Rift Valley fever (2006–2009) shown by red, blue and green dots over eastern and southern Africa and the Indian Ocean islands.

At a gross global scale, the *El Niño* phase of ENSO causes distinct and simultaneous patterns of flooding and drought (Fig. 1). Specifically, there is a tendency for wetter-than-normal conditions and floods to occur over Eastern Africa, the southern tier of the United States, Southern Brazil/northern Argentina and eastern-and-central Pacific Islands, Ecuador, and Peru. Similarly, there is a tendency for drought to occur over a large area of Southeast Asia, Australia, northern and north–eastern Brazil, and Southern Africa. These conditions are largely reversible during the *La Niña* phase of ENSO [16], [20], [21]. Such extremes in regional climate regimes can create ecological conditions that influence the emergence or re-emergence of mosquito vectors, their distribution and abundance (as well as those of their vertebrate hosts), their population dynamics, and the transmission of mosquito-borne diseases of global public health relevance [1], [22]. Climate-disease teleconnection studies have been carried out, but most have been limited by poor reporting and a lack of geo-referenced disease databases. In this study we analyze and illustrate how recent outbreaks of two mosquito-borne diseases, chikungunya and Rift Valley fever [8], [9], [22]–[24], over Africa and the western Indian Ocean basin islands were coupled to specific climate anomaly patterns. We use geo-referenced disease occurrence data (Fig. 2), and explore spatial and temporal associations of these disease outbreaks and how they change with variability in the underlying climate and ecological patterns.

## Materials and Methods

To determine the ecological and climatic conditions leading to and associated with Rift Valley fever and chikungunya mosquito-borne disease outbreaks, we analyzed relationships between locations of outbreaks and patterns of change in vegetation, rainfall, and temperature.

### Data

#### Rift Valley fever and chikungunya disease data

The baseline disease case location data used in this study were based on human and livestock epidemiological surveys by different institutions in various countries. These data are limited to outbreaks between 2006–2009 for Rift Valley fever in East Africa, Sudan, and Southern Africa. For chikungunya data, we compiled data from two sources. The first source was data from the recent epidemic in Eastern Africa and Western Indian Ocean islands, covering 2004–2006. The second source was a historical 1952–2010 dataset compiled from various sources, including literature in the online archives of the U S Centers for Disease Control and Prevention (CDC), the WHO, and other relevant sources to create a statistically robust sample for this study. We were primarily concerned with geographic locations of the disease cases in order to compare them with environmental data. At each historical outbreak location an approximate geographic latitude/longitude location was determined using place names. Historical outbreaks were located in East Africa, Central Africa, South Asia (primarily India and Bangladesh), and Southeast Asia, providing a broader historical context in which to analyze chikungunya-climate relationships. Most comprehensive geo-referenced records of chikungunya were limited to the recent epidemic period (2004–2010). Full details of chikungunya and Rift Valley fever case data are provided in sections 3 and 5 of Text S1.

#### Environmental and climate data

We utilized a number of environmental and climate data sets described in detail in Text S1 and summarize in Table 1. Satellite-derived Africa Rainfall Climatology (ARC) rainfall estimates for Africa and global surface air temperature and rainfall data from National Centers for Environmental Prediction and the National Centre for Atmospheric Research (NCEP/NCAR) were used to examine the climate anomaly patterns. The concept of teleconnections was illustrated using the Global Precipitation Climatology Project (GPCP) data set. SPOT Vegetation normalized difference vegetation index (NDVI) data were used as a proxy for ecological dynamics to assess and infer ecological conditions associated with Rift Valley fever outbreaks.

**Table 1:** Climate and disease data sets used in the study.

Data	Source	Coverage	Climatology period	Purpose
NINO 3.4 SST	NOAA/CPC	5°N–5°S, 170°W–120°W, monthly	1971–2000	teleconnections
WIO	NOAA/CPC	10°N–10°S, 40°–64°E, monthly	1971–2000	teleconnections
GPCP Rainfall	NOAA/CPC	global, monthly, 1°	1979–2009	teleconnections, chikungunya
NCEP Air Temperature	NOAA/CPC	global, monthly, 2.5°	1968–1996	chikungunya
ARC Rainfall	NOAA/CPC	Africa, monthly, 10 km spatial resolution	1995–2006	Rift Valley fever, chikungunya
SPOT Vegetation AVHRR NDVI	VITONASA/GIMMS	Africa, monthly, 1 km, 8 km spatial resolution	May 1998–April 2008	Rift Valley fever
Disease Data (Rift Valley fever, chikungunya)	CDC-K, WHO, FAO, OIE and various national governments	episodic (Rift Valley fever: 2006–2009, chikungunya: 1952–2010)	N/A	climate-ecology-disease teleconnections and relationships

All anomaly indices were computed as monthly departures from their respective climatological values (long-term means) defined by the periods shown above. NINO 3.4 SST index was computed by the National Oceanic and Atmospheric Administration Climate Prediction Center (NOAA/CPC) as part of operational ENSO monitoring activities. We computed the WIO index directly from the global SST data based on previous research by Linthicum et al. (1999). SPOT Vegetation data were processed by Vlaamse Instelling voor Technologisch Onderzoek (VITO) in Belgium into 10-day composite data. Monthly composites, long-term means, and anomalies from these data were processed by the NASA/Global Inventory Modeling and Mapping Studies (GIMMS) group.

### Approach

We mapped disease location data on corresponding NDVI and climate data anomalies in order to understand associations between specific anomaly patterns in ecological and climate variables and disease outbreak patterns through space and time. We further explored the associations by plotting and comparing disease data against cumulative rainfall and vegetation index anomalies to illustrate the lag time between the driving climate conditions and the timing of first case disease occurrence for Rift Valley fever. For chikungunya we further investigated the relationships among surface air temperature, precipitation anomalies, and chikungunya outbreaks through correlation analysis. For Rift Valley fever we further investigated relationships between precipitation and Rift Valley fever outbreaks through logistic regression. Results were interpreted in terms of vector biology and population dynamics. We suggested caveats for non-outbreak years when climate and ecological conditions would indicate an imminent outbreak.

### Mapping and analysis

#### Anomaly calculations

**Figure 3:**
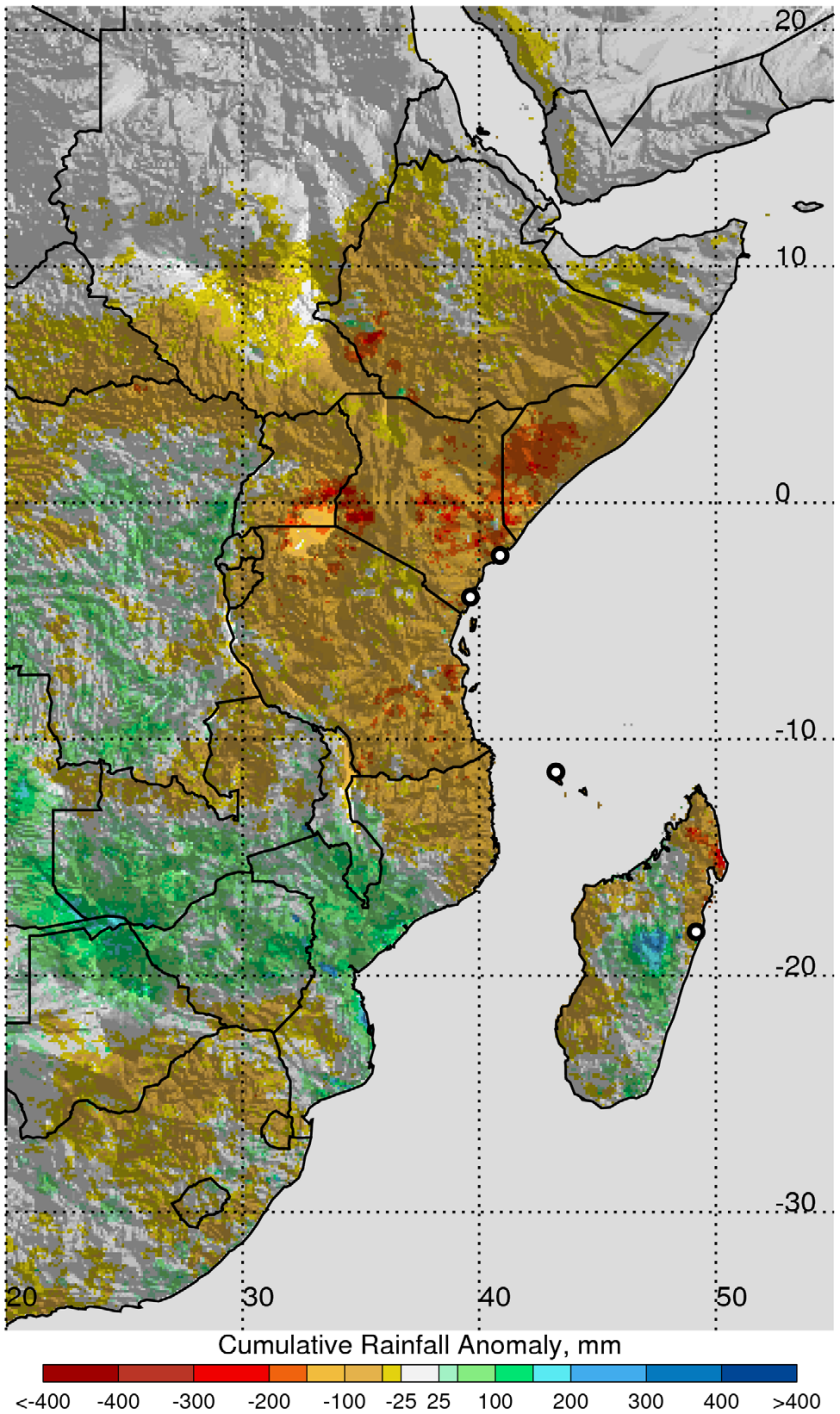
Cumulative rainfall anomalies over Eastern Africa, October–December, 2005. Negative rainfall anomalies correspond to the large-scale regional drought in Eastern Africa during October–December, 2005. Anomalies were calculated with reference to the 1995–2000 long term mean. Epicenters of chikungunya outbreaks during this period are shown by the four open black dots.

**Figure 4:**
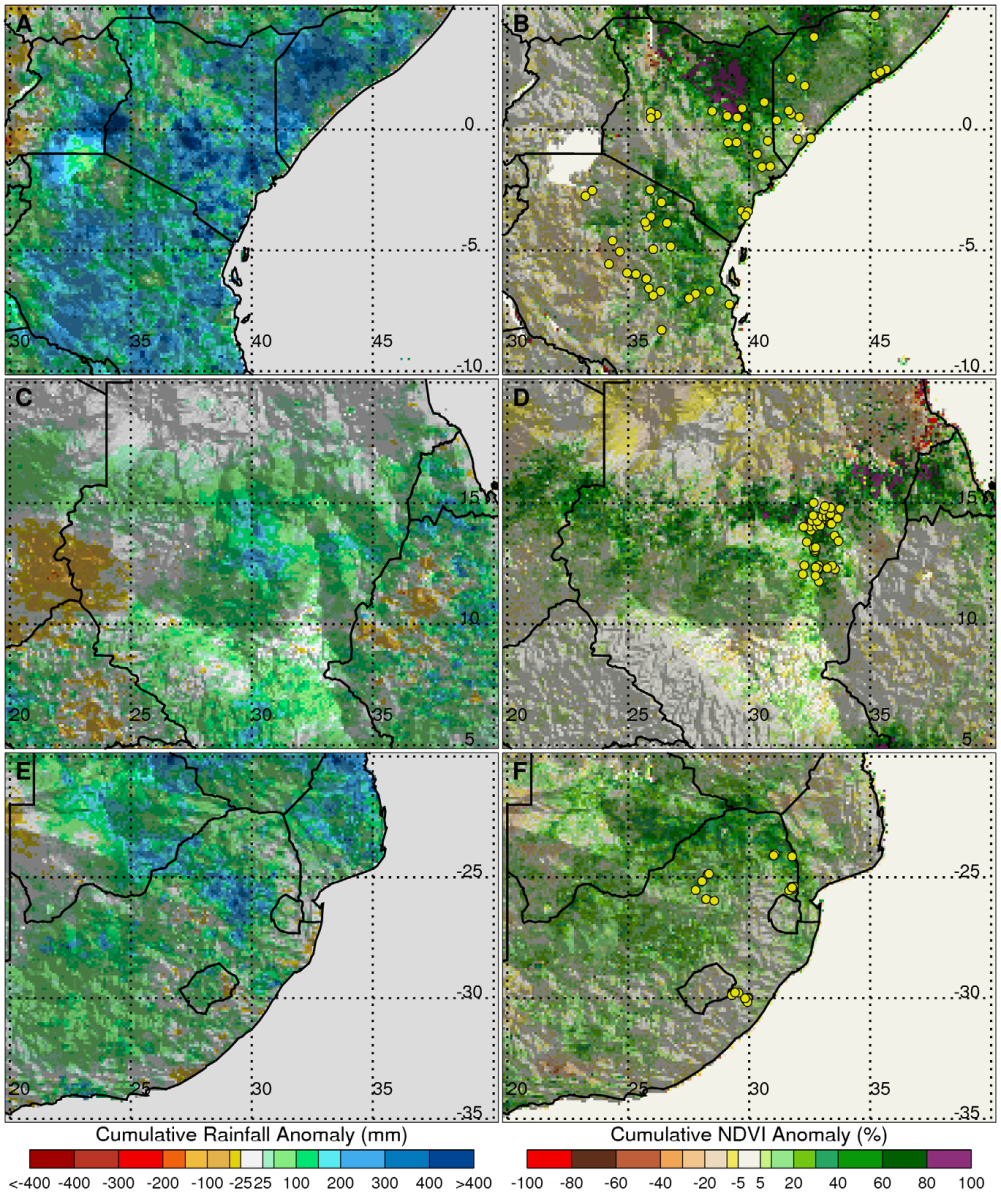
Cumulative rainfall anomalies and vegetation index anomalies for East Africa, Sudan and Southern Africa. Patterns of rainfall anomalies preceding outbreaks of Rift Valley fever in (A) East Africa: September–December, 2006, (C) Sudan: June–September, 2007, and (E) Southern Africa: October, 2007–January, 2008. Each outbreak was preceded by persistent and above-normal rain on the order of +200 mm for a period of ∼2–4 months (Fig. S2). This resulted in anomalous green-up of vegetation, creating ideal ecological conditions for the production of *Aedes* and *Culex* mosquito vectors that transmit Rift Valley fever virus to domestic animals and humans. Vegetation anomalies are shown for (B) East Africa: October, 2006–January 2007, (D) Sudan: July–September, 2007, and (F) Southern Africa: October, 2007–January, 2008. Rift Valley fever outbreaks are marked with yellow dots.

In general, for each climate/environmental variable, we calculated anomalies as follows:
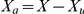
where *X_a_* = was the anomaly or difference of climate variable for any given month (*X*) from its long-term mean (*X_u_*) The anomaly was a quantitative measure of the departure from normal/average conditions resulting in, for example using rainfall (Figs. 3 and 4), extreme wetness (above-normal rainfall, positive anomaly values) or extreme dryness (drought, negative anomaly values).

#### Teleconnections mapping

To illustrate the concept of teleconnections globally we calculated monthly rainfall anomalies for the GPCP data set based on 1979–2008 long term means. The rainfall anomalies were then correlated with the NINO3.4 sea surface temperature (SST) anomaly index by calculating Pearson's correlation coefficient over the monthly time series to produce the map shown in Fig. 1. Additional details of teleconnection analyses are provided in section 1 of Text S1.

#### Time-space mapping

**Figure 5:**
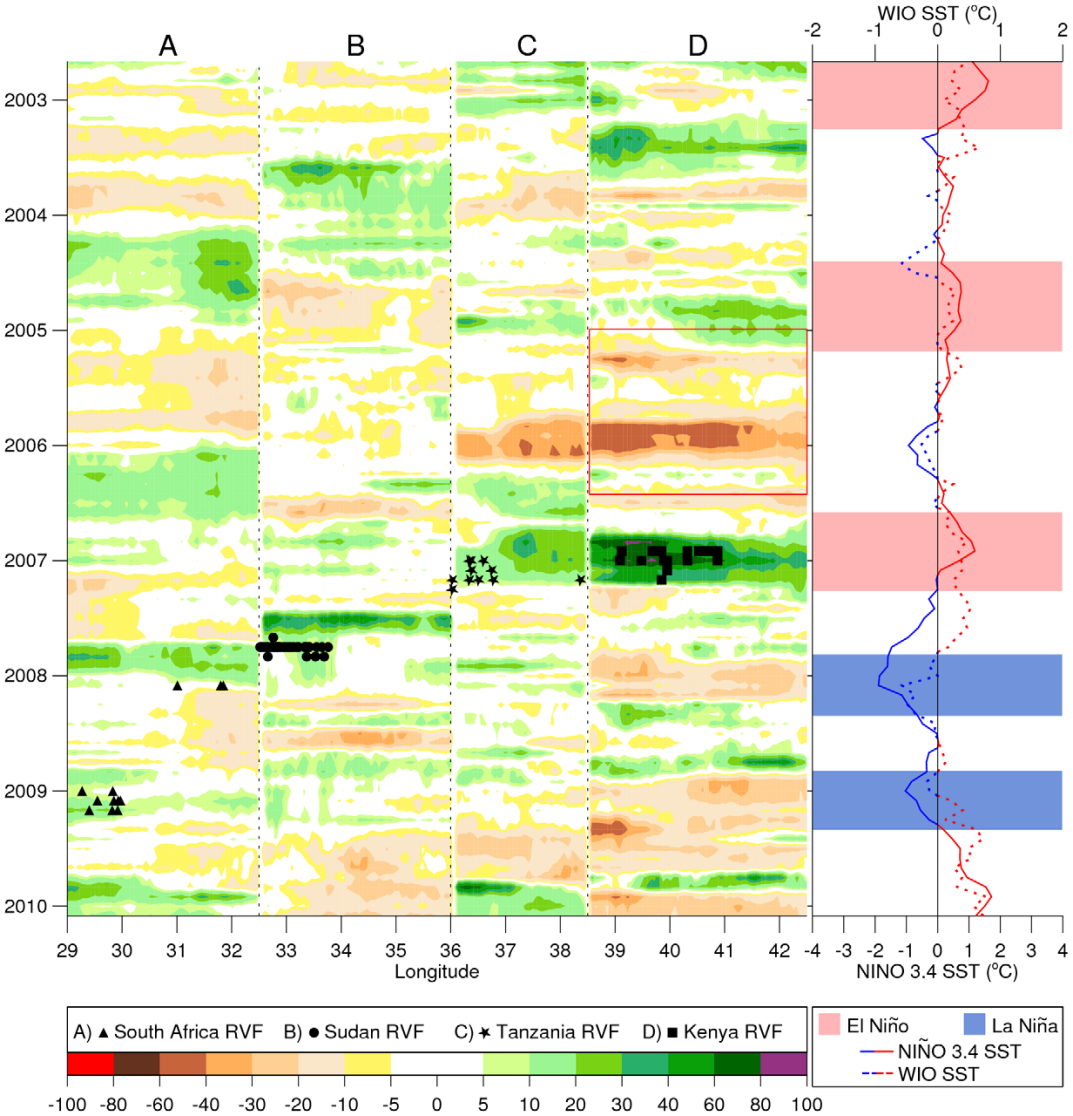
Spatial and temporal anomaly patterns of NDVI, SST in relation to RVF outbreaks. Spatial and temporal anomaly patterns in normalized difference vegetation index for selected areas of South Africa (A: 29°E and 32.5°E, averaged from 23°S to 27°S), Sudan (B: 32.5°E and 34°E, averaged from 11°N to 15°N), Tanzania (C: 34°E and 37°E, averaged from 4.5°S to 8.5°S) and Kenya (D: 37°E and 42.5°E, averaged from 2°S to 2°N). Regions were plotted by geographic position west to east and represent areas with dense concentrations of Rift Valley fever cases. NDVI anomalies are depicted as percent departures from the 2002–2008 long-term mean, and show the response of vegetation to variations in rainfall. Periods shaded in green to purple indicate above-normal vegetation conditions associated with above-normal rainfall. Periods of persistent drought or below normal rainfall are shown in shades of yellow to red. Each Rift Valley fever outbreak was preceded by above-normal vegetation conditions resulting from persistent above-normal rainfall in the Horn of Africa and Sudan in 2006–2007. Chikungunya epidemics occurred over East Africa and Indian Ocean islands during the 2005–2006 drought period shown by negative NDVI anomalies from 2005–2006 [D: red boxed area]. Clusters of epidemics/epizootics of Rift Valley fever in East Africa (2006–2007) and Sudan (2007) occurred during the *El Niño* event of 2006–2007 when there were concurrent anomalously warmer WIO and Nino 3.4 SSTs. The transition to *La Niña* conditions in late 2007–early 2008 spatially shifted the area of above-normal rainfall and enhanced vegetation conditions to South Africa and Madagascar between February–March, 2008 and sporadically between February–March, 2009 in South Africa, leading to outbreaks of Rift Valley fever in these regions. This illustrates that spatial displacements in extreme rainfall and ecological conditions driven by large-scale climate mechanisms such as ENSO and regional circulation lead to spatial-temporal shifts in areas at risk for outbreaks of these mosquito-borne diseases.

To illustrate the ecoclimatic teleconnection connection patterns in relation to Rift Valley fever outbreaks we plotted a Hovmöller diagram of NDVI anomalies for each outbreak region (East Africa, Sudan, and South Africa) against the NINO3.4 SST anomalies. This diagram shows the spatial and temporal dynamics of NDVI anomalies in relation to temporal dynamics of ENSO (represented by the NINO 3.4 index), Western equatorial Indian Ocean (WIO) SST anomalies, and the Rift Valley fever outbreak patterns. Details of how the Hovmöller diagram was derived are given in section 6 of Text S1 and the results are presented in Fig. 5.

#### Cumulative rainfall analysis

Since Rift Valley fever outbreaks are known to follow periods of extended above-normal rainfall, we calculated a cumulative rainfall anomaly index based on the ARC data set as follows:
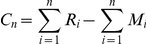
where *C_n_* is the cumulative rainfall anomaly value for time steps 1 to *n*, Σ is the summation function, *R_i_* was total rainfall at time step *i* of the series, and *M_i_* is the average total rainfall for time step *i*. The time period was normally chosen to represent the rainy season, in which case *C_n_* measured the difference between the current and average seasonal total rainfall [15]. To determine whether the ARC estimates indicated flood conditions, i.e., where *C_n_* was consistently positive, we calculated *C_n_* for the months prior to reported Rift Valley fever outbreaks for four regions: East Africa (outbreak period December, 2006 to April, 2007), Sudan (September–November, 2007), Southern Africa (January–April, 2008), and Madagascar (March–May, 2008). A similar method was applied to NDVI data for outbreak periods for East Africa (2006–2007), Sudan (2007), and South Africa (2007–2008, 2008–2009), and for the initial chikungunya outbreak period in East Africa (2004–2006). This index represents a proof of concept first suggested by Linthicum et al. [25], that Rift Valley fever activity follows widespread and above-normal rainfall enabling the emergence of large populations of Rift Valley fever vector mosquito species. Further details of cumulative rainfall analysis are provided in section 4 of Text S1.

#### Logistic regression

In order to quantify the relationship between rainfall anomaly and the occurrence of Rift Valley fever, we used logistic regression. For each region (East Africa, Sudan, Southern Africa, Madagascar), we calculated the cumulative rainfall anomaly for the four months immediately prior to and including the onset of Rift Valley fever activity. These regional reference periods were as follows: (1) East Africa, September–December, 2006, for the December, 2006 outbreak; (2) Sudan, June–October, 2007, for the October, 2007 outbreak; (3) South Africa, October, 2007–January, 2008, for the January, 2008 outbreak; and (4) Madagascar, December, 2007–March, 2008, for the March, 2008 outbreak. For each outbreak site given in Fig. 1, we calculated the cumulative rainfall anomaly over the regional reference period for each year 2004–2008. The cumulative rainfall anomaly was expressed as a fraction of mean cumulative rainfall to make the regression coefficients independent of the rainfall unit of measurement. We coded Rift Valley fever presence with a 1 for the year of outbreak and absence with a 0 for all other years, and then ran a logit model regression of Rift Valley fever presence/absence on cumulative rainfall anomaly. The logit model was
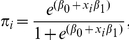
where *π_i_* was the code for disease absence/presence for observation *i* (1 or 0), *x_i_* was the cumulative rainfall anomaly (expressed as a fraction of mean cumulative rainfall) for observation *i*, and the fitted coefficients, shown in Table 2, were *β*_0_ (intercept term) and *β*_1_ (rainfall anomaly term).

**Table 2:** Results of logistic regression of Rift Valley fever presence/absence on cumulative rainfall anomalies.

Region		coefficients	std error	z value	p(>|z|)	confidence
East Africa (n = 383)	*β*_0_	−3.1202	0.3050	−10.231	<2×10^−16^	>99.9%
	*β*_1_	2.8096	0.3264	8.608	<2×10^−16^	>99.9%
Sudan (n = 257)	*β*_0_	−4.6153	0.8952	−5.156	2.53×10^−7^	>99.9%
	*β*_1_	26.5603	5.1397	5.168	2.53×10^−7^	>99.9%
South Africa (n = 185)	*β*_0_	−2.022	0.260	−7.777	7.40×10^−15^	>99.9%
	*β*_1_	4.575	1.106	4.135	3.55×10^−5^	>99.9%
Madagascar (n = 65)	*β*_0_	−2.0897	0.5849	−3.573	0.000353	>99.9%
	*β*_1_	−10.6091	3.8901	−2.727	0.006387	99.9%

Logistic regression of Rift Valley fever presence/absence on cumulative rainfall anomalies over a 4 month period. For each region, the top row presents results for the intercept term (*β*_0_) and the bottom row (*β*_1_) for the rainfall anomaly term. Regional Rift Valley fever outbreaks were significantly positively correlated with persistently above-normal cumulative rainfall over a 4 month period (99.9% confidence, *β*_1_>0), except in Madagascar (*β*_1_<0).

#### Correlation analysis

**Figure 6:**
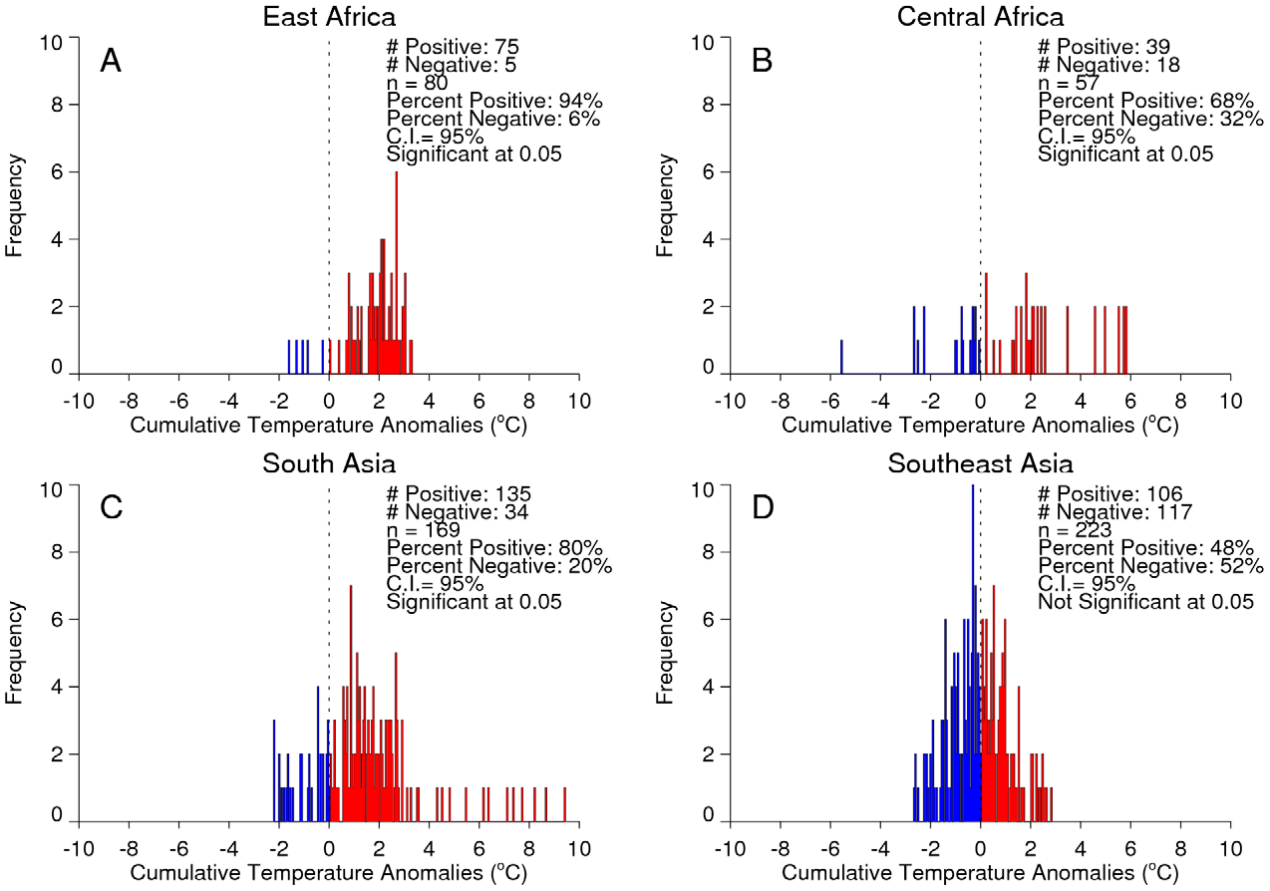
Frequency distributions of chikungunya outbreak events in relation to temperature. Frequency distributions of chikungunya outbreak events and 4-month cumulative temperature anomalies for East Africa (A), Central Africa (B), South Asia (C), and Southeast Asia (D). The 4-month anomaly threshold was used to represent periods of either cool temperatures or drought and extreme high temperatures The dashed line at zero depicts the 1979–2009 long-term mean temperature, with warmer-than-normal temperatures shown to the right (red) and cooler-than-normal temperatures shown to the left (blue) of the line. Cases shown to the right of the dashed line occurred during periods of elevated temperature with a persistence of 4 months.

**Figure 7:**
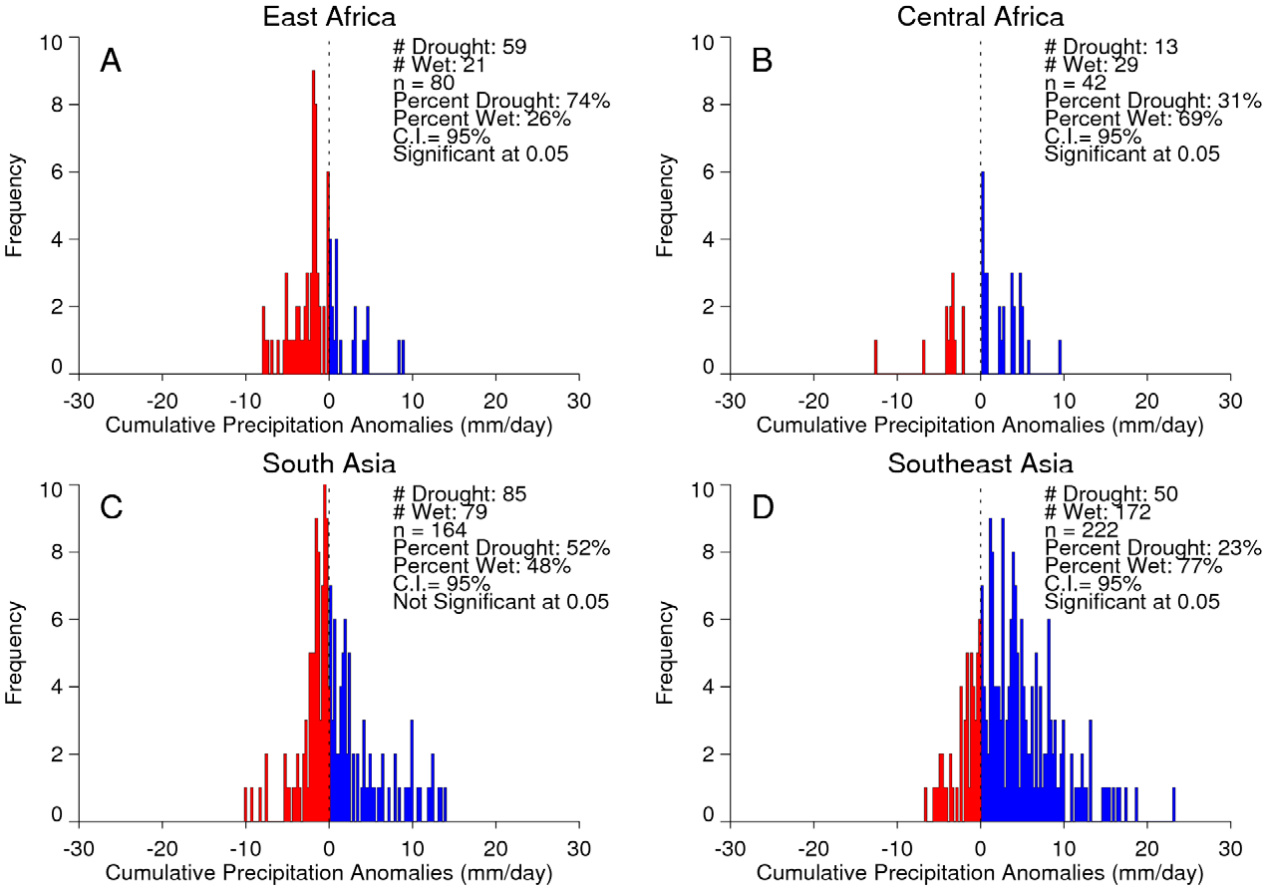
Frequency distributions of chikungunya outbreak events in relation to precipitation. Frequency distributions of chikungunya outbreak events and 4-month cumulative precipitation anomalies for East Africa (A), Central Africa (B), South Asia (C), and Southeast Asia (D). The 4-month anomaly threshold was used to represent periods of either persistent above-normal rainfall/wetness or persistent drought conditions. The dashed line at zero depicts the (1979–2009) long term rainfall, with greater-than-normal precipitation shown to the right (blue) and lower-than-normal precipitation shown to the left (red) of the line. Cases shown to the left of the dashed line occurred during periods of drought with a persistence of 4 months.

For chikungunya, we tested the hypothesis that disease outbreaks were correlated with elevated temperature and/or drought by plotting occurrences against surface air temperature anomalies and precipitation anomalies under two scenarios. In the first scenario, meant to simulate high temperatures and moderate drought, cumulative anomalies were made for a 3-month period prior to and then including the actual month of the case, for a total of 4 months. An example for April would be: January, February, March, and April anomalies aggregated to create the 4-month cumulative anomaly. Using the same method, a second scenario was constructed for a prolonged period of high temperatures and severe drought, except this time for a 7-month total. A frequency of occurrence was obtained by extracting values from each of the two scenarios for each of the chikungunya locations, for each region. For the temperature anomalies, the occurrences were classified as higher-than-normal if the anomalies were >0, or cooler-than-normal if <0. The precipitation anomalies were classified similarly as wetter-than-normal or drought if >0 or <0, respectively. Because each geographic region had an occurrence sample size of ≥30, the binomial test of significance was used. This test assumes a normal distribution, random sampling, and mutually exclusive data. A confidence level of 95% was assumed for the 2-tailed test. If the region's occurrences passed the test of significance, the nature of the relationship was tested by calculating the Pearson's correlation coefficient. Additional details of chikungunya correlation analyses are provided in sections 2 and 5 of Text S1. Results by region are presented in Figs. 6 and 7.

## Results

### Climate-disease teleconnections

The map in Fig. 1 shows the correlation between monthly NINO3.4 SST anomalies and monthly GPCP rainfall anomalies for 1979–2010. It illustrates how variations in sea surface temperature in the equatorial eastern Pacific (ENSO) influence rainfall variability at various locations around the world. It is also a depiction of the concept of teleconnections. The central to eastern Pacific Ocean region, Ecuador, Peru, Eastern equatorial Africa, and the southern tier of the United States indicate the tendency for wetter-than-normal conditions in these regions during the *El Niño* (warm) phase of ENSO. Negative correlations over Southern Africa and the western Pacific region, including Australia, the greater Indonesian Basin, and northern South America, indicate the tendency for drier-than-normal conditions during *El Niño* [10]. These anomaly patterns are largely reversed during the *La Niña* (cold) phase of ENSO as shown in previous studies [10]. Extremes in rainfall anomalies resulting from phase shifts in ENSO affect regional ecological patterns [11], [12] differentially, and influence the emergence of different disease vectors and consequently patterns of vector-borne disease outbreaks [13], [14]. In particular, there is a tendency for outbreaks of Rift Valley fever to occur in Eastern Africa during the *El Niño* phase and for outbreaks of Rift Valley fever to occur in Southern Africa during the *La Niña* phase, as will be shown later in this study.

### Temporal sequence of outbreaks in relation to eco-climatic conditions

The 2004–2009 period of analysis is an illustration of the above teleconnection patterns and demonstrates how different climate and ecological anomaly extremes resulting from these teleconnections influence vector-borne disease outbreaks through variations in temperature, rainfall, and ecology. The clusters of outbreaks and epidemics/epizootics of chikungunya and Rift Valley fever across Africa (Fig. 2) [8], [9], [23], [24] occurred during periods of severe drought for chikungunya (2004–2006; Fig. 3) [19], [26], and during periods of above-normal rainfall for Rift Valley fever (late 2006 to 2009, Fig. 4) [8], [27]. During 2004–2006 there was a regional chikungunya epidemic covering coastal East Africa that expanded to cover western Indian Ocean islands, and later parts of India and Southeast Asia through 2010 (Fig. S4). The bulk of the human cases were reported during the most severe drought period of 2005–2006 in East Africa and the western Indian Ocean islands. This chikungunya outbreak was consistent with drought resulting from circulation anomalies forced by the ENSO cold phase (*La Niña*) and a regional circulation pattern characterized by anomalously high pressure in the western equatorial Indian Ocean and low pressure in the east. These pressure patterns led to enhanced surface westerlies, causing enhanced descending motion over East Africa and the western Indian Ocean and ascending motion over Indonesia [21], [26], [27]. This pattern of regional circulation hampered convective activity and precipitation and resulted in one of the most severe droughts ever observed in the region (Fig. 2).

Historically, chikungunya is known to be enzootic in west and central Africa in a sylvatic cycle involving wild non-human primates and forest species of *Aedes* mosquitoes [28]. The first and subsequent documented outbreaks of chikungunya occurred during periods of heavy rainfall, filling natural and artificial containers that serve as immature mosquito habitats [29], [30]. However, in 2004–2006 the severe drought described above [26] led to widespread storage of water in containers around households. Unprotected stored water allowed *Aedes aegypti* on the East African coast [9], [31] to reproduce in large numbers and permit establishment of a Central/East African genotype of chikungunya virus in highly populated areas of the East African coast and the western Indian Ocean islands [28]. In addition and possibly most importantly, elevated temperatures during the drought facilitated the amplification of the virus and increased vectorial capacity in the mosquitoes [9], [31]. Exposure of *Ae. aegypti* larvae to similarly elevated temperatures in the laboratory has been shown to select for strains adapted to survive longer at these temperatures and significantly increase susceptibility of adult mosquitoes to chikungunya virus, enhancing vectorial capacity and vector competence, respectively [32]. This suggests *Ae. aegypti* was adaptable to actual environmental conditions of severe drought and elevated temperatures presented here. The initial chikungunya cases were identified at health care facilities in Lamu (June, 2004) in the initial stages of the drought, and Mombasa (November, 2004) as the drought peaked in Kenya (Fig. 3).

In time the chikungunya outbreak spread and impacted the western Indian Ocean islands including Seychelles, Comoros, Mayotte, Mauritius, and La Reunion, all in 2005, infecting between 30–75% of the populations in affected areas [2], [21] (Figs. 2 and 3). The entire western equatorial Indian Ocean region was impacted by a large scale drought in 2005 (Fig. 3) [26]. In December, 2005 a mutation was observed in some of the chikungunya isolates from La Reunion that caused a single amino acid substitution in the E1 envelope glycoprotein (E1-A226V) [33], [34]. Although *Aedes albopictus* was considered a competent but secondary vector of chikungunya [35], the E1-A226V mutation increased virus replication and dissemination in this mosquito, enhancing its vector competence and increasing the potential for the virus to permanently extend its geographic range [33], [34]. As many as 3 other independent events led to the E1-A226V mutation in India, Cameroon, Gabon, and Sri Lanka, and geographical expansion of the adaptation of the virus to *Ae. albopictus* [36], [37], [38]. Subsequently the outbreak continued eastward affecting many Indian Ocean countries and in conjunction with human travel and tourism affected some European countries ([9], [24], [33], Text S1).

Cessation of the drought in eastern Africa was marked by the development of contrasting patterns of SST anomalies in the equatorial Indian Ocean, with positive SST anomalies in the equatorial western sector and negative SST anomalies in the eastern sector, and a warm ENSO event in the eastern Pacific Ocean. These patterns of SST anomalies (Fig. S1) and associated precursor pressure anomalies led to enhanced easterlies, fast southern trade winds and ascending motion over East Africa, and descending motions over Southeast Asia [19], [26], [27]. The combined effect of these conditions resulted in persistent, above-normal, and widespread rainfall in excess of 200 mm per month during September–December, 2006 in endemic regions of Eastern Africa (Fig. 4A) and severe drought conditions in Southeast Asia. The above-normal rainfall flooded low lying wetlands known as *dambos* [39], the primary habitats of *Aedes mcintoshi* mosquitoes pre-infected with Rift Valley fever virus from prior epizootics. The abnormally high and sustained precipitation led to not only rapid emergence and development of the mosquitoes, but also enhanced and sustained survival because of rapid emergence of protective vegetation habitat, as indicated by large positive NDVI anomalies (Fig. 4B) [10], [23]. Large increases in numbers of virus-carrying mosquitoes in northeastern Kenya and southern Somalia quickly followed, and the first human Rift Valley fever cases were identified from serological surveys in mid-December, 2006 in Kenya and Somalia.

The enhancement of *El Niño* and WIO positive SST conditions and then the shift in the main rainfall belt southwards into Tanzania resulted in a shift of the area at risk from Rift Valley fever activity southwards into Tanzania (Fig. 4A). The first cases of Rift Valley fever in humans were reported in Tanzania in February 2007 [23], [40]. While the Pacific NINO3.4 region shifted to a cold phase with the emergence of *La Niña* conditions in May, 2007, the western equatorial Indian Ocean (Fig. S2) continued to warm with SST anomalies as high as +1°C between May–July, 2007. These conditions led to enhanced convective activity across the Sahel region with persistent and above-normal rainfall centred over central Sudan between June–October, 2007 (Fig. 4C). As in East Africa, these anomalous rains flooded *dambo* habitats and areas within the Gezira irrigation scheme as shown by the positive NDVI anomalies (Fig. 4D) resulting in the emergence of large numbers of Rift Valley fever-carrying *Aedes* and *Culex* mosquitoes. The first cases of Rift Valley fever in humans and livestock in Sudan were reported in mid-October, 2007. This was the first reported outbreak of Rift Valley fever in Sudan since 1976, although serologic evidence suggests an outbreak also occurred in 1981 [41], [42].

The shift from *El Niño* conditions to *La Niña* (Fig. S2) conditions in the summer and fall of 2007 moved the main area of enhanced rainfall from Eastern to Southern Africa (Fig. 4E). The above-normal and widespread rainfall between October, 2006–February, 2007 resulted in flooding and anomalous green-up of vegetation in Rift Valley fever-endemic areas of Southern Africa (Fig. 4F) and Madagascar. Cases of Rift Valley fever in livestock and humans were identified in mid-February, 2008 and subsequently in some farm workers and veterinary students in South Africa. Human cases of Rift Valley fever were identified in Madagascar over a large part of the country from March–May, 2008 [23]. The persistence of *La Niña* conditions and enhanced westerlies in the equatorial Indian Ocean through mid-2009 [20] resulted in recent and continuing severe drought conditions in East Africa and continued above-normal rainfall conditions in Southern Africa [43]. As a result there was a recurrence of Rift Valley fever activity in Southern Africa during the southern hemisphere summer period through 2011. Figure 5 summarizes the temporal and spatial anomaly patterns in vegetation (i.e., ecology) as they relate to the temporal patterns in SST anomalies and the resulting temporal distribution patterns of Rift Valley fever outbreaks for East Africa, Sudan, and Southern Africa during this period.

### Comparison of chikungunya and Rift Valley fever with climate anomalies

An analysis of the January, 1979–February, 2010 relationships between chikungunya activity and surface air temperature anomalies and precipitation anomalies is shown in Figs. 6 and 7, respectively. The figures show the frequency distribution of the number of reported chikungunya outbreak events against rainfall and temperature anomalies. Persistent temperature anomalies over a four month period were classified as hot if anomalies were >0 or cool if <0. Persistent precipitation anomalies over a 4 month period were classified as drought if <0 and wet if >0. In East Africa, Central Africa, and South Asia, 94%, 68%, and 80% of the outbreaks occurred during warmer-than-normal temperatures, and these differences were significant at *p*<0.05 (Figs. 6A–C). However, in Southeast Asia 52% of the outbreaks occurred during cooler-than-normal temperatures (Fig. 6D). In East Africa outbreaks were also significantly positively correlated with drought conditions at *p*<0.05 (Fig. 7A), and not significantly correlated in South Asia (Fig. 7C). In Central Africa (Fig. 7B) and in Southeast Asia (Fig. 7D) outbreaks were significantly negatively correlated with drought at *p*<0.05. The positive correlation between chikungunya outbreaks and warmer-than-normal temperatures in Africa and South Asia was consistent with non-sylvatic transmission by *Ae. aegypti* and *Ae. albopictus* in highly populated domestic settings where domestic and peri-domestic stored water supplies were the likely source of the mosquitoes [9], [32], [33], [44], [45].

The relationship between precipitation and Rift Valley fever was determined through a logistic regression of Rift Valley fever presence/absence on cumulative precipitation anomalies for the 4 months immediately preceding each Rift Valley fever outbreak [7], [8] (Fig. S3; Text S1). Results by region (East Africa, Sudan, South Africa, and Madagascar) are presented in Table 2. For all 4 regions a significant relationship was found between cumulative rainfall anomalies and Rift Valley fever presence with at least 99.9% confidence. For East Africa, Sudan, and South Africa this relationship was strongly positive (*β*_1_>0), with the highest cumulative rainfall anomalies yielding the highest odds of Rift Valley fever presence, equivalent to an outbreak, as indicated by the positive rainfall anomaly terms estimated in Table 2. This relationship confirmed experimental findings by Linthicum et al. [46] that persistent, widespread, and above-normal rainfall is required to flood *dambo* habitats in order to create ideal conditions to spawn abundant mosquito populations on a large scale that would result in a Rift Valley fever epizootic. As shown in Fig. S3, each of the selected outbreak locations for each region was preceded by above-normal rainfall for 3–4 months before the first case of Rift Valley fever, which would likely create ideal ecological conditions for an increase in Rift Valley fever mosquito vector emergence and survival.

In contrast, for Madagascar a negative relationship was found (*β*_1_<0), with the model predicting higher odds of Rift Valley fever outbreaks when rainfall was less than normal. Although the 2008 Madagascar Rift Valley fever outbreak was initially triggered by rainfall [23], [47], the subsequent spread of the outbreak appears to have been related to the introduction of infected livestock. In Madagascar, livestock located in heavy rainfall areas in the south had become infected with Rift Valley fever and were then transported to other parts of the country to the north. Even though rainfall was only slightly above normal in these northern areas, precipitation was sufficient to produce abundant *Culex* populations originating from flooded domestic and semi-domestic immature mosquito breeding habitats. The *Culex* mosquitoes, efficient vectors of Rift Valley fever, then transferred the virus from the newly arrived infected livestock to surrounding human populations.

## Discussion

We have shown that inter-annual climate variability, as expressed by the ENSO phenomenon in association with regional climatic circulation mechanisms in the equatorial Indian Ocean, had broad influence on two mosquito-borne disease outbreaks over the greater Eastern Africa region, Southern Africa, and western Indian Ocean islands through opposite spatial shifts in precipitation and vegetation anomaly patterns (Figs. 1, 4, and 5). In general, above-normal rainfall, cooler-than-normal temperatures, and above-normal vegetation development were strongly associated with the ecology of Rift Valley fever outbreaks, and drought and warmer-than-normal temperatures were associated with chikungunya epidemics in the greater East African region.

Historically, large scale outbreaks of chikungunya have been in large highly populated urban settings of tropical Asia, transmitted by *Ae. aegypti*, and in highly populated areas in Africa with smaller outbreaks limited to rural areas [30]. Current evidence as shown in this paper, however, illustrates that recent outbreaks in East Africa and western Indian Ocean islands occurred in coastal urban centres with large population densities [9], [24], [31] (Fig. S4). Additionally, the recent outbreaks in Gabon in 2010 have been in urban and suburban settings [48] and have occurred during a period of elevated temperature and drought as recently as May, 2010. This suggests that highly vector competent *Ae. aegypti* and *Ae. albopictus* exist in Africa and the greater Indian Ocean region [44]. Another property of historical chikungunya outbreaks was association with above-normal rainfall, such as the 1952–53 epidemic in Tanzania where excess rainfall in 1952 likely contributed to a spill over of the virus from a sylvan cycle involving *Aedes furcifer/taylori* and non-human primates to *Ae. aegypti* and humans. Yet, our findings here show that recent outbreaks, at least in East Africa and the western Indian Ocean islands, were favoured by extended and severe drought coupled with elevated temperature conditions [9], [26], [27] (Fig. 3). This suggests that *Ae. aegypti* has adapted well to urban settings in Africa, as it has in much of the tropics, sub-tropics, and temperate regions of the world, after its origin in sylvan Africa in the absence of human populations [45], [49], [50]. This also implies that *Ae. aegypti* has adapted to regional climate conditions in the western Indian Ocean region that were on average dry and rainfall-deficient compared to Southeast Asia [19].

In South Asia the occurrence of the majority of chikungunya cases during elevated temperatures during both rainy and drought periods strongly suggests that temperature alone was a major driving factor, involving both urban and rural transmission by *Ae. aegypti* and *Ae. albopictus*, respectively. On the other hand, the primary vector in Southeast Asia, *Ae. albopictus* is endemic and highly adapted to the prevailing wet climate regime that is its preferred habitat. As we have demonstrated here, this climate regime is primarily associated with rainfall and not elevated temperatures. This is consistent with rural transmission by *Ae. albopictus*, and may suggest the possibility of a sylvan cycle [51]. Although chikungunya, transmitted by *Ae. aegypti* largely disappeared from India and Southeast Asia in the late 1970s–early 1980s [52], [53], both Asian and African genotypes are currently sympatric there [54]. The extent and scale of the 2004–2006 chikungunya epidemic in Eastern Africa was perhaps a revelation of Jupp and McIntosh's [30] hypothesis more than two decades ago that population growth in Africa with the associated urbanization would lead to epidemics on a scale similar to those experienced in Asia.

We have also illustrated that there was a spatial shift in the area at risk of Rift Valley fever activity from Eastern to Southern Africa in tandem with a phase shift from *El Niño* to *La Niña* conditions in the eastern Pacific Ocean and SST anomalies in the western Indian Ocean (Fig. 5). Anomalously heavy, widespread, and prolonged rainfall events (Fig. 4) that caused Rift Valley fever outbreaks in domestic animals and humans in East Africa and Sudan (2006–2007) and Southern Africa (2008–2009) followed a transition of the ENSO phenomenon from the warm *El Niño* phase (2006–2007) to the cold *La Niña* phase (2007–2009), and the resultant displacement of the center of above-normal rainfall from Eastern to Southern Africa. This elevated rainfall flooded mosquito habitats that introduced the virus into domestic animal populations by producing large numbers of vertically infected *Ae. mcintoshi* and other *Aedes* species mosquitoes. Sustained elevated rainfall then triggered production of large populations of *Culex* mosquitoes that served as secondary vectors of Rift Valley fever to animals and humans [10], [23]. As our findings here indicate, Rift Valley fever outbreaks occurred during the short-rains seasons for East Africa (October–December), Sudan (October–November), and Southern Africa (December–February). During some years, this period occurred when there were enhanced equatorial easterlies that led to above-normal rainfall and flood conditions over the eastern landmass of Africa [26], [27]. This pattern was particularly enhanced when it was in phase with the warm episode of ENSO (Fig. 5; [8], [10], [21], [26]).

Our analyses of the 2006–2009 Rift Valley fever outbreaks confirmed that there was a very close correlation between outbreaks and persistent (i.e., 3–4 months) above-normal rainfall [46], [55]. The only known exception to this in sub-Saharan Africa was the man-made flooding of mosquito habitats associated with the damming of the Senegal River in 1987 that led to the large 1987–1988 outbreak in Senegal and Mauritania in the absence of excessive rainfall [56]. In Egypt, outbreaks appear to be related to rainfall in the upper regions of the Nile in Uganda, Ethiopia, and Sudan that results in flooding of habitats along the lower Nile in Egypt. In Madagascar, rainfall precipitated the 2008 outbreak in the southern part of the country, but the majority of human and animal cases actually occurred in other parts of the country where ample *Culex* mosquito vectors in domestic settings efficiently transmitted the virus to animals and humans. Still, there may be periods of heavy rainfall and ideal ecological conditions that do not result in Rift Valley fever outbreaks (Fig. 5). This be may due to (1) livestock and human population immunity, factors not fully understood, (2) changes in land use, e.g., the transformation of *dambos* into agricultural land, (3) flooding events or the timing, pattern, and distribution of rainfall that may not be consistent enough to support sufficient batch hatching of multiple generations of vectors to result in an outbreak, and (4) failure to detect disease outbreaks due to weak surveillance systems in sub-Saharan Africa, particularly in the livestock/agricultural sector. In addition, only in the last 10 years have georeferenced disease databases begun to be compiled, and often the data are spotty and usually only gathered after an epidemic/epizootic has occurred. This type of outbreak response reporting misses cases that occur in less severe climate and disease conditions and thus largely go unreported.

### Conclusions

Our knowledge of teleconnection events and the quasi-cyclical nature of climate variability may allow parts of Africa, the Indian Ocean basin islands, and elsewhere within the greater tropics to have more than a year warning prior to Rift Valley fever outbreaks [23] and other humanitarian climate-related conditions, permitting more precise targeting of vaccine strategies, mosquito control, animal quarantine, and public education strategies. Additionally, identifying the potential for Rift Valley fever outbreaks to occur in Africa and controlling these outbreaks will be of interest to regions of the world that are not currently endemic for Rift Valley fever. The documented expansion of the range of Rift Valley fever beyond sub-Saharan Africa into Egypt in 1977 [56] and into the Arabian Peninsula in 2000 [57], [58], [59] makes Rift Valley fever a likely candidate for further expansion and a significant threat to most of the world where immunologically naïve animal species and humans exist, and competent *Culex* or *Aedes* vector mosquito species are present. Furthermore, there is a significant economic threat from Rift Valley fever in non-endemic countries. For example, the U.S. had beef related exports in 2003 of $5.7 billion and should Rift Valley fever occur in the U.S. the OIE will impose a 4 year trade ban until free of the disease for 6 months [60]. The climate-disease teleconnections examined here can also be used to better understand the temporal dynamics of the burden of other diseases such as dengue, malaria, or cholera which can be influenced by major climate events [11], [12].

Outbreaks of mosquito-borne diseases on epidemic scales, such as those experienced during 2005–2009 in Africa and the western Indian Ocean islands, place a huge burden on public healthcare systems and the economy. Outbreaks of chikungunya are also an impediment to tourism, a major contributor to the gross national product of countries and island nation states in the region. The costs to the economies of East Africa in lost trade in livestock due to Rift Valley fever outbreaks were estimated to be $65 million [61] during the 2006–2007 outbreak. Furthermore, there is extended disruption in trade due to livestock movement bans following an outbreak. Given the greater risk of spread and recurrence of outbreaks of these diseases, it is critical that countries in the region have the capacity to anticipate the changing and variable nature of the climate in the region to prevent or minimize the emergence and re-emergence of such diseases. Therefore there is need for public health authorities to take advantage of climate observations and analyses in times of extreme climate variability to aid response and mitigation planning including vector surveillance and control, vaccination, and public education in areas that may be impacted by disease outbreaks. In addition, climate-based predictions offer opportunities for virologists, epidemiologists, entomologists, physicians, and veterinarians to understand the biological and cyclic nature of the disease and how its episodic occurrence relates to livestock immunity in recently infected areas, and the potential for re-emergence of the disease in livestock and human populations.

It is apparent from our analyses that in changing and variable climate, arboviruses and their mosquito vectors are going to adapt to the existing climatic and ecological conditions in a new region, and the resultant disease transmission will vary accordingly and may not be the same manifestation as observed in the original endemic regions. Combining satellite-derived measurements and analyses of climate and ecology with an understanding of mosquito vector biology and human and animal population immunity status can contribute substantially towards reducing the global burden of vector-borne diseases.

### Ethics statement

All data analyzed were anonymized. We only used GPS latitude-longitude coordinates for cases, and we did not handle or deal with any human or animal specimens.

## Supporting Information

Text S1 **Supporting information and methods: detailed data sources, processing, and analysis.**
(DOC)

Figure S1 **SST anomalies during the peak of the** ***El Niño*** **event from December, 2006–February, 2007.** Global seasonal equatorial sea surface temperature anomalies during the peak of the *El Niño* event December, 2006–January, 2007.
(TIFF)

Figure S2 **SST anomalies during the peak of the** ***La Niña*** **event from December, 2007–February, 2008.** Global seasonal equatorial sea surface temperature anomalies during the peak of the *La Niña* event December, 2007–February, 2008.
(TIFF)

Figure S3 **Cumulative daily rainfall profiles for periods of Rift Valley fever activity for selected outbreak sites.** Cumulative daily rainfall (green lines) profiles for periods of Rift Valley fever activity and mean long-term cumulative daily rainfall (red lines) for sites with reported Rift Valley fever activity. Dotted line represents when the first case of Rift Valley fever was identified at each location. Each of the outbreak locations was preceded by above-normal rainfall for 3–4 months.
(TIFF)

Figure S4 **Distribution of 2004–2010 chikungunya outbreaks in relation to human population density.** Each symbol represents the year(s) when an outbreak was reported at a specific geographic location. Most chikungunya activity has occurred in locations with high population densities (>500 people per square kilometre).
(TIF)
