# Supplementary material for: Climate Teleconnections and Recent Patterns of Human and Animal Disease Outbreaks
Source: PLoS Negl Trop Dis. 2012 Jan 24;6(1):e1465. doi: 10.1371/journal.pntd.0001465 (PMC3265456; doi:10.1371/journal.pntd.0001465)
Supplement: Text S1 — Supporting information and methods: detailed data sources, processing, and analysis. (DOC) [file pntd.0001465.s001.doc]

SUPPORTING INFORMATION AND METHODS:

**DETAILED DATA SOURCES, PROCESSING, AND ANALYSIS**

1. **Global Climate Teleconnection Patterns**: The global *El Niño*/Southern Oscillation (ENSO) teleconnections map (Fig. 1) was derived by calculating per-pixel correlations between the NINO3.4 sea surface temperature (SST) anomaly index, drawn from the central-eastern Pacific Ocean region (5°N-5°S) (170°W-120°W), and global rainfall anomalies**.** NINO3.4 SST is one of the four SST regions (as well as the Southern Oscillation Index [SOI]) used by the National Oceanographic and Atmospheric Administration’s Climate Prediction Centre (NOAA/CPC) to monitor the phase, amplitude and propagation of ENSO events (see <http://www.cpc.noaa.gov/products/analysis_monitoring/ensostuff/nino_regions.shtml>). The SST data on which the NINO3.4 index is based are a blend of direct observations from ships, buoys, satellite imagery from National Oceanic and Atmospheric Administration (NOAA) Advanced Very High Resolution Radiometer (AVHRR) instruments, and SSTs simulated by sea-ice cover. The monthly optimum interpolated fields were derived by a linear interpolation of the weekly fields to daily fields, and then averaging daily values over a month based on Smith and Reynolds [62]. NOAA-calculated NINO SST indices are publicly available at: <http://www.cpc.noaa.gov/data/indices/>. Figures S1 and S2 show the seasonal SST anomaly patterns during the peak of the *El Niño* event from December, 2006-January, 2007 and during the peak of the *La Niña* event from December, 2007-January, 2008, respectively. These variations in SST anomaly patterns influence the departure patterns in rainfall over specific regions (Fig. 1), ultimately affecting the ecology and populations of vectors and disease outbreak patterns**.**

The base rainfall data used in the correlation mapping are known as the Global Precipitation Climatology Project (GPCP) data set. The GPCP was established by the World Climate Research Program to address the problem of quantifying the distribution of precipitation around the globe over many years. The data set combines precipitation measurements from several sources including Special Sensor Microwave/Imager data from the Defence Meteorological Satellite Program and infrared (IR) precipitation estimates computed from geostationary satellites (United States, Europe, and Japan), and secondarily from polar-orbiting satellites (United States).

In addition, other estimates were derived from the Atmospheric Infrared Sounder data, originating from the NASA Aqua platform, and the NOAA polar orbiting satellite series provided data via the Television Infrared Observation Satellite Program Operational Vertical Sounder and the Outgoing Longwave Radiation Precipitation Index. Rain data were assembled and analyzed by the Global Precipitation Climatology Centre of the Deutscher Wetterdienst and by the Climate Prediction Center of NOAA. These multi-source rainfall estimates were blended together to produce the necessary global gridded precipitation fields and the blended data set covers January, 1979-present (with some delay). Details of the data set are described in [63] and can be found at: <http://precip.gsfc.nasa.gov/gpcp_v2.1_comb_new.html>. We calculated monthly rainfall anomalies based on 1979-2008 long-term means. The rainfall anomalies were then correlated with the NINO3.4 SST anomaly index by calculating Pearson’s correlation coefficient over the monthly time series to produce the teleconnections map shown in Fig. 1.

The teleconnections map shows areas that are influenced by ENSO phase changes. Positive correlations over equatorial Africa (covering part of the study region), the central to eastern Pacific Ocean region, Ecuador, Peru, and the southern tier of the United States indicate the tendency for wetter-than-normal conditions in these regions during the *El Niño* (warm) phase of ENSO. Negative correlations over the western Pacific region including Australia, the greater Indonesian Basin, Southern Africa, and northern South America indicate the tendency for drier-than-normal conditions during *El Niño* [64]. These patterns are largely reversed during the *La Niña* (cold) phase of ENSO as previously shown by [64]. These extremes in the rainfall anomaly patterns as a result of phase shifts in ENSO affect regional ecological patterns [65,66] differentially and influence the emergence of different disease vectors and consequently patterns of vector-borne disease outbreaks [67,68].

1. **NCEP/NCAR Reanalysis Temperature Data:** The surface air temperature data used to compute temperature anomalies came from the National Centers for Environmental Prediction and the National Center for Atmospheric Research (NCEP/NCAR) Reanalysis 1 dataset. The purpose of the dataset is to provide a long-term data record of surface climate variables by using analysis and forecast data assimilation techniques [69]. The NCEP/NCAR Reanalysis data were obtained from the Earth System Research Laboratory Physical Sciences Division Data found at <http://www.esrl.noaa.gov/psd/>. The monthly mean and long-term monthly mean surface temperature data with coverage from 90°N-90°S and 180°E-180°W and a spatial resolution of 2.5° was obtained from 1948-present. The long-term monthly mean surface temperatures were computed using a base period of 1968-1996. Anomalies were computed by subtracting the long-term monthly mean from each month, respectively. Given the drought 2004-2006 period in East Africa coinciding with the chikungunya outbreak, these data were used to examine temperature conditions during chikungunya outbreaks for both Eastern Africa and Asia.
2. **Geographic Distribution of Rift Valley Fever and Chikungunya over Africa and the Western Indian Ocean Islands:** Fig. 2 shows spatial distribution patterns of recent epidemics/epizootics and outbreaks of vector-borne diseases (Rift Valley fever and chikungunya) from 2004-2009. The baseline disease outbreak data were based on epidemiological surveys by different institutions in various countries and included both human and livestock data. Chikungunya case data were collected by a combined team of personnel from the Division of Disease Surveillance and Response, Ministry of Health, Kenya; US Army Medical Research Unit, Nairobi, Kenya; Kenya Medical Research Institute, Global Disease Detection Division of the Centers for Disease Control-Kenya; and Global Alert and Response Department (HSE/GAR) of the World Health Organization from surveys at hospital facilities in the vicinity of the outbreaks in both Kenya and western Indian Ocean Islands [70].

Rift Valley fever human-case data were collected by the same agencies in Kenya. For Sudan, Somalia, and Tanzania the data were collected by the respective ministries of health in collaboration with the World Health Organization. For Madagascar the disease data were collected by a team from Institut Pasteur de Madagascar and the World Health Organization. The data from South Africa were collected by the Ministry of Agriculture and Special Pathogens Unit, National Institute for Communicable Diseases, and included locations of Rift Valley fever livestock cases [71,72]. All case data were collected using global positioning system devices that register the location by latitude and longitude of each disease incidence; these coordinates were mapped to produce Fig. 1. These disease incidence data are a rich information source for examining relationships among climate variability, ecological dynamics, and patterns of disease outbreaks that have not been possible in the past. Continued collection of such geo-referenced data will be critical to understanding impacts of a changing climate on disease incidence through space and time.

1. **Rainfall and Vegetation.** To illustrate and map conditions preceding and during Rift Valley fever outbreaks we computed daily cumulative rainfall and monthly normalized difference vegetation index (NDVI) anomalies during the target period for each outbreak. The rainfall data were sourced from NOAA/CPC Africa rainfall climatology (ARC) archives. These rainfall estimates were produced daily using a combination of satellite inputs and rain gauge measurements. As with GPCP, the ARC data were derived from several satellites and *in situ* sources, including: the polar orbiting Special Sensor Microwave/Imager and Advanced Microwave Sounding Unit microwave sensors; infrared bands of the geostationary METEOSAT platforms; and rain gauge measurements from the Global Telecommunications System daily total rainfall product [73]. The ARC data were mapped to a spatial resolution of 0.1° latitude and longitude over Africa.

Previous field research [74] has shown that Rift Valley fever virus-carrying mosquito vectors require above-normal and widespread rainfall to flood *dambo* habitats over a period of not less than 50 days [74,75,76]. These conditions can be illustrated by the use of the cumulative rainfall anomalies function as described in the Materials and Methods [76]. Cumulative anomalies were calculated and plotted for selected outbreak locations in East Africa (outbreak period December, 2006-April, 2007), Sudan (September-November, 2007), Southern Africa (January-April, 2008), and Madagascar (March-May, 2008). All regions and selected sites of outbreaks had positive cumulative rainfall anomalies for the measured periods in the areas where outbreaks were reported, with the largest rainfall surplus in East and Southern Africa, and moderately wet conditions in Sudan. These positive cumulative rainfall anomalies prior to the reported Rift Valley fever outbreaks indicated flood conditions over potential *dambo* habitats [74,75,76]. Fig. S3 shows profiles of cumulative rainfall patterns for selected outbreak locations over these regions.

However, while flooding is a necessary condition for the hatching of Rift Valley fever virus-infected mosquito eggs, the survival of these vectors is only possible under appropriate ecological conditions. Satellite-derived NDVI data [77] can be used as a proxy for the complex of ecological conditions, including ambient temperature, soil moisture, and habitat that would guarantee emergence, survival, and propagation of these vectors [78,79]. In order to estimate the ecological suitability for vector survival, we computed cumulative NDVI anomalies as the difference between current and mean total NDVI, expressed as a percentage of the mean total. The cumulative NDVI percent anomalies for each region are given in Figs. 4B (East Africa: October, 2006-January, 2007), 4D (Sudan: July-September, 2007), and 4F (Southern Africa: October, 2007-January, 2008). NDVI anomalies exceeding 60% (dark green and purple colors) were found in East Africa, Sudan, and Southern Africa, indicating conditions were much greener than normal in these regions. These maps illustrate that each Rift Valley fever outbreak was preceded by above-normal rainfall and anomalous green-up in vegetation. To quantify the relationship between rainfall anomaly and the presence of Rift Valley fever, we used logistic regression as explained in the Materials and Methods.

1. **Historical Chikungunya Outbreak Data and Analysis:** Historical 1952-2010 chikungunya outbreak data were compiled from on-line literature archives of the Centers for Disease Control and Prevention, the World Health Organization, and other relevant sources [80-108]. At each outbreak location an approximate geographic latitude/longitude point was estimated using place names if a georeference was not provided by the source. According to our inventory, historical outbreaks were located in the regions of East Africa, Central Africa, South Asia (primarily India and Bangladesh), and Southeast Asia. Regional records were then processed in sequence to make them mutually exclusive both spatially and temporally. To eliminate auto-correlations, clusters of data were delineated in 2.5° resolution boxes and then replaced with a single randomly selected sample from the cluster. The sample period was limited to January, 1979-February, 2010, because this was the common temporal time period across the 3 datasets. In order to form a background population for the purpose of performing statistical analysis, locations of no outbreaks were selected from 1979-present using a random point generator within the predetermined boundaries of each region. The boundaries delineated for East Africa were 25°S-11°N and 30°E-50°E, Central Africa from 17°S-20°N and 8°E-30°E, South Asia from 5°N-30°N and 68°E-93°E, and Southeast Asia from 10°S-22°N and 95°E-127°E. The ratio used for presence: absence of an outbreak for each region was 3:1. Month and years for the background population were first chosen systematically; then, by using a random generator drawing from the collected archive of chikungunya outbreak cases as a basis, we tested the hypothesis that disease outbreaks were correlated with elevated temperature and/or drought by plotting occurrences against: (1) surface air temperature anomalies (Fig. 6), and (2) precipitation anomalies (Fig. 7) under two scenarios as explained in the Materials and Methods.

Lastly, the locations of chikungunya cases were mapped on population density. A population density dataset was obtained from the Socioeconomic Data and Applications Center at Columbia University, current as of 2005. For the purposes of this research we subset the region impacted by chikungunya covering Africa, the Mediterranean, and the Indian Ocean. All chikungunya outbreak sites were color-coded by the year(s) of outbreak and plotted on the population density data for the subset area in Fig. S4. Most of the sites in Asia were at village level, which led to clustering. It can be noted that, unlike Rift Valley fever, most if not all cases of chikungunya have occurred in coastal towns or urban settlements with population densities >500 people per square kilometre. Such dense population concentrations created conditions for explosive epidemics.

1. **Spatio-temporal Climate-Ecology-Disease Teleconnection Patterns:** In order to illustrate the continental-scale climate teleconnections and their relationships to patterns of ecological variability and disease outbreak patterns over Africa, we derived a Hovmöller time-space diagram (Fig. 5). This diagram allowed us to capture and map the space and time dynamics of the propagating anomaly patterns in vegetation or ecological dynamics. The data used in this analysis consisted of monthly composite NDVI from September 2002-February 2010 derived from the AVHRR on the NOAA-17 polar-orbiting satellite. NDVI was calculated according to [77]. These data were processed by the Global Inventory Monitoring and Mapping Studies group at NASA’s Goddard Space Flight Center. Monthly data for Africa mapped at 8 km spatial resolution were created using the maximum value compositing procedure to reduce cloud contamination, topographic, and angular effects [109]. We calculated long-term monthly means of NDVI using 2002-2008 as the reference mean period and calculated percent anomalies according to [110]. The percent anomalies were defined as the current month minus the long-term mean of that month divided by the mean of that month, and then multiplied by 100. The resulting values expressed NDVI departures in percentage format.

To plot the complex Hovmöller diagram, bounding boxes for specific areas were defined based on epicentres or the dense clusters of Rift Valley fever outbreak locations in each region. For Southern Africa the area covers 29ºE-32.5ºE, and we averaged anomalies from 23.0ºS-27.0ºS. For Sudan, the area covers 32.5ºE-36.0ºE, and we averaged anomalies from 11.0ºN-16.0ºN. Tanzania was defined by the region between 36.0ºE-38.5ºE, and with anomalies averaged from 4.0ºS-8.0ºS. Finally, for Kenya, the bounding region was between 38.5ºE-42.5ºE, and we averaged anomalies between 0.0ºN-4.0ºN. For each pixel within the bounding box, percent anomaly NDVI data were averaged over the latitudinal direction. The data were organized by geographic location according to longitude from west to east, with overlapping values between regions removed. Rift Valley fever outbreak cases were plotted at the approximate longitude location by time of case identification for each of the specific regions. NINO3.4 Index was obtained from the National Weather Service’s Climate Prediction Center for the same time period and plotted in degrees Celsius.

We calculated and plotted the Western Indian Ocean (WIO) sea surface temperature anomaly index in degrees Celsius for the same time period. Previous research has shown that episodic outbreaks of Rift Valley fever in Eastern Africa were driven by *El Niño* (warm NINO3.4 SST) and warmer-than-normal SSTs in the western equatorial Indian Ocean region (WIO) [76,78]. The space-time NDVI anomaly diagram shows: (1) the variability in vegetation over these regions through time, and how these patterns of variability were coupled to the variations in NINO3.4 and WIO sea surface temperature anomaly patterns, and (2) the patterns of disease outbreaks through time and space. However, there were periods when ecological conditions were primed for outbreaks to occur and no Rift Valley fever activity was reported, for example January-May, 2006, February-August, 2004 (South Africa), September-December, 2003 (Sudan), and March-June, 2003 (Kenya). This may be because of: (1) livestock and human population immunity, factors not fully understood or studied; (2) changes in land use, in particular the transformation of *dambos* into agricultural land; (3) flooding events or the pattern and distribution of the rainfall that may not have been consistent enough to support sufficient batch hatching of multiple generations of vectors to result in an outbreak; and (4), likely the most common, the failure to detect disease outbreaks or low level virus circulation due to weak surveillance systems in sub-Saharan Africa, particularly in the livestock agricultural sector.

**REFERENCES**

1. Smith TM, Reynolds RW (2004) Improved extended reconstruction of SST (1854-1997). J Clim 17: 2466-2477.
2. Adler RF, Huffman GJ, Chang A, Ferraro R, Xie PP, et al. (2003) The version-2 Global Precipitation Climatology Project (GPCP) monthly precipitation analysis (1979-present). J Hydrometeorol 4: 1147-1167.
3. Ropelewski CF, Halpert MS (1987) Global and regional scale precipitation patterns associated with the *El Niño*/Southern Oscillation. Mon Weather Rev 115: 1606-1626.
4. Anyamba A, Eastman JR (1996) Interannual variability of NDVI over Africa and its relation to *El Niño*/Southern Oscillation. Int J Remote Sens 17: 2533-2548.
5. Myneni RB, Los SO, Tucker CJ (1996) Satellite-based identification of linked vegetation index and sea surface temperature anomaly areas from 1982-1990 for Africa, Australia, and South America. Geophys Res Lett 23: 729-732.
6. Glantz MH (1991) Introduction. In: Glantz MH, Katz RW, Nicholls N, editors. Teleconnections Linking Worldwide Climate Anomalies. New York: Cambridge University Press. pp. 1-12.
7. Anyamba A, Chretien J-P, Small J, Tucker CJ, Linthicum KJ (2006) Developing climate anomalies suggest potential disease risks for 2006-2007. Int. J Health Geogr 5: 60, doi: 10.1186/1476-072X-5-60 <http://www.ij-healthgeographics.com/content/5/1/60/abstract>
8. Kalnay EC, Kanamitsu M, Kistler R, Collins W, Deaven D, et al. (1996) The NCEP/NCAR 40-year reanalysis project. Bull Amer Meteorol Soc 77: 437-471.
9. Chretien J-P, Anyamba A, Bedno SA, Breiman RF, Sang R, et al. (2007) Drought-associated chikungunya emergence along coastal East Africa. Am J Trop Med Hyg 76: 405-407.
10. WHO (2007) Outbreaks of Rift Valley fever in Kenya, Somalia and United Republic of Tanzania, December 2006-April 2007. Weekly Epidemiological Record 82: 169-180.
11. Anyamba A, Linthicum KJ, Small J, Britch SC, Pak E, et al. (2010) Prediction, assessment of the Rift Valley fever activity in East and Southern Africa 2006-2008 and possible vector control strategies. Am J Trop Med Hyg 83(S2): 43-51.
12. Love TB, Kumar V, Xie P, Thiaw W (2004) A 20-year daily Africa precipitation climatology using satellite and gauge data. In: Proceedings of the 84^th^ American Meteorological Society Annual Meeting, P5.4. Conference on Applied Climatology 11-15 January 2004, Seattle, WA.
13. Linthicum KJ, Davies FG, Bailey CL, Kairo A (1984) Mosquito species encountered in a flooded grassland dambo in Kenya. Mosquito News 44: 228-232.
14. Davies FG, Linthicum KJ, James AD (1985) Rainfall and epizootic Rift Valley fever. Bull World Health Organ 63: 941-943.
15. Anyamba A, Chretien JP, Small J, Tucker CJ, Formenty PB, et al. (2009) Prediction of a Rift Valley Fever Outbreak. Proc Natl Acad Sci U S A 106: 955-959, doi:10.1073/pnas.0806490106.
16. Tucker CJ (1979) Red and photographic infrared linear combinations for monitoring vegetation. Rem Sens Environ 8: 127-150.
17. Anyamba A, Linthicum KJ, Mahoney R, Tucker CJ, Kelley PW (2002) Mapping potential risk of Rift Valley fever outbreaks in African savannas using vegetation index time series data. Photogramm Eng Remote Sens 68: 137-145.
18. Linthicum KJ, Bailey CL, Davies FG, Tucker CJ (1987) Detection of Rift Valley fever viral activity in Kenya by satellite remote sensing imagery. Science 235: 1656-1659.
19. Robinson MC (1955) An epidemic of virus disease in Southern Province, Tanganyika Territory, in 1952-53. I. Clinical features. Trans R Soc Trop Med Hyg 49: 28-32.
20. Shah KV, Gibbs CJ Jr, Banerjee G (1964) Virological investigation of the epidemic of haemorrhagic fever in Calcutta: isolation of three strains of chikungunya virus. Indian J Med Res 52: 676-683.
21. Carey DE, Myers RM, Deranitz CM, Jadhav M, Reuben R (1969) The 1964 chikungunya epidemic at Vellore, South India, including observations on concurrent dengue. Trans Roy Soc Trop Med Hyg 63: 434-445.
22. Rodrigues FM, Patankar MR, Banerjee K, Bhatt PN, Goverdhan MK, et al. (1972) Etiology of the 1965 epidemic of febrile illness in Nagpur city, Maharashtra State, India. Bull World Health Organ 46: 173-179.
23. Padbidri VS, Gnaneswar TT (1979) Epidemiological investigations of chikungunya epidemic at Barsi, Maharashtra state, India. J Hyg Epid Microb Im 23: 445-451.
24. Saluzzo JF, Gonzalez JP, Herve JP, Georges AJ (1980) Epidemiological study of arboviruses in the Central African Republic: demonstration of chikungunya virus during 1978 and 1979. Bull Soc Pathol Exot Filiales 73: 390-399.
25. Thaikruea L, Charearnsook O, Reanphumkarnkit S, Dissomboon P, Phonjan R, et al. (1997) Chikungunya in Thailand: A re-emerging disease? Southeast Asian J Trop Med Public Health 28: 359-364.
26. Retuya TJA Jr, Ting DL, Dacula BD, Lanada JM, et al. (1998) Chikungunya fever outbreak in an agricultural village in Indang, Cavite, Philippines. Philippine J Microbiol Infect Dis 27: 93-96.
27. Nur Y, Groen J, Heuvelmans H, Tuynman E, Copra C, et al. (1999) An outbreak of West Nile fever among migrants in Kisangani, Democratic Republic of Congo. Am J Trop Med Hyg 61: 885-888.
28. Lam SK, Chua KB, Hooi PS, Rahimah MA, Kumari S, et al.(2001) Chikungunya infection – an emerging disease in Malaysia. Southeast Asian J Trop Med Public Health 32: 447-451.
29. Pastorino B, Muyembe-Tamfum JJ, Bessaud M, Tock F, Tolou H, et al. (2004) Epidemic resurgence of chikungunya virus in Democratic Republic of Congo: Identification of a new Central African strain. J Med Virol 74: 277-282.
30. Porter KR, Tan R, Istary Y, Suharyono W, Sutaryo, et al. (2004) A serological study of chikungunya virus transmission in Yogyakarta, Indonesia; Evidence for the first outbreak since 1982. Southeast Asian J Trop Med Public Health 35: 408-415.
31. Laras K, Sukri NC, Larasati RP, Bangs MJ, Kosim R, et al.(2005) Tracking the re-emergence of epidemic chikungunya virus in Indonesia. Trans Roy Soc Trop Med Hyg 99: 128-141.
32. Peyrefitte CN, Rousset D, Pastorino BAM, Pouillot R, Bessaud M, et al. (2006) Chikungunya virus, Cameroon, 2006. Emerg Infect Dis. 13: 768-771.
33. AbuBakar S, Sam IC, Wong PF, Matrahim N, Hooi PS, et al. (2007) Re-emergence of endemic chikungunya, Malaysia. Emerg Infect Dis 13: 147-149.
34. Jain SK, Kumar K, Bhattacharya D, Venkatesh S, Jain DC, et al. (2007) Chikungunya virus disease in district Bhilwara (Rajasthan) India. J Commun Dis 39: 25-31.
35. Manimunda SP, Singh SS, Sugunan AP, Singh O, Roy S, et al. (2007) Chikungunya fever, Andaman and Nicobar Islands, India. Emerg Infect Dis 13: 1259-1260.
36. Noridah O, Paranthaman V, Nayar SK, Masliza M, Ranjit K, et al. (2007) Outbreak of chikungunya due to virus of Central/East African genotype in Malaysia. Med J Malaysia 62: 323-328.
37. Sergon K, Yahaya AA, Brown J, Bedja SA, Mlindasse M, et al. (2007) Seroprevalence of chikungunya virus infection on Grande Comore Island, Union of the Comoros, 2005. Am J Trop Med Hyg 76: 1189-1193.
38. Beesoon S, Funkhouser E, Kotea N, Spielman A, Robich RM (2008) Chikungunya fever, Mauritius, 2006. Emerg Infect Dis 14: 337-338.
39. Kaur P, Ponniah M, Murhekar MV, Ramachandran V, Ramachandran R, et al. (2008) Chikungunya outbreak, South India, 2006. Emerg Infect Dis 14: 1623-1625.
40. Ratsitorahina M, Harisoa J, Ratovonjato J, Biacabe S, Reynes JM, et al. (2008) Outbreak of dengue and chikungunya fevers, Toamasina, Madagascar, 2006. Emerg Infect Dis 14: 1135-1137.
41. Sergon K, Njuguna C, Kalani R, Ofula V, Onyango C, et al. (2008) Seroprevalence of chikungunya virus (CHIKV) infection on Lamu Island, Kenya, October 2004. Am J Trop Med Hyg 78: 333-337.
42. Sissoko D, Malvy D, Giry C, Delmas G, Paquet C, et al. (2008) Outbreak of chikungunya fever in Mayotte, Comoros archipelago, 2005-2006. Trans Roy Soc Trop Med Hyg 102: 780-786.
43. Kannan M, Rajendran R, Sunish IP, Balasubramaniam R, Arunachalam N, et al. (2009) A study on chikungunya outbreak during 2007 in Kerala, South India. Indian J Med Res 129: 311-315.
44. Leroy EM, Nkoghe D, Ollomo B, Nze-Nkogue C, Becquart P, et al. (2009) Concurrent chikungunya and dengue virus infections during simultaneous outbreaks, Gabon, 2007. Emerg Infect Dis 15: 591-593.
45. Ng L-C, Tan LK, Tan CH, Tan SSY, Hapuarachchi HC, et al. (2009) Entomologic and virologic investigation of chikungunya, Singapore. Emerg Infect Dis 15: 1243-1249.
46. Samuel PP, Krishnamoorthi R, Hamzakoya KK, Aggarwal CS (2009) Entomo-epidemiological investigations on chikungunya outbreak in the Lakshadweep Islands, Indian Ocean. Indian J Med Res 129: 442-445.
47. Thavara U, Tawatsin A, Pengsakul T, Bhakdeenuan P, Chanama S, et al. (2009) Outbreak of chikungunya fever in Thailand and virus detection in field population of vector mosquitoes, *Aedes aegypti* and *Aedes albopictus* Skuse (Diptera: Culicidae). Southeast Asian J Trop Med Public Health 40: 951-962.
48. Holben BN (1986) Characteristics of maximum-value composite images from temporal AVHRR data. Int J Remote Sens 7: 1417-1434.
49. Anyamba A, Tucker CJ, Mahoney R (2002) *El Niño* to *La Niña*: Vegetation response patterns over East and Southern Africa during the 1997-2000 period. J Clim 15: 3096-3103.
