# Supplementary figures and images for: Climate Teleconnections and Recent Patterns of Human and Animal Disease Outbreaks
Source: PLoS Negl Trop Dis. 2012 Jan 24;6(1):e1465. doi: 10.1371/journal.pntd.0001465 (PMC3265456; doi:10.1371/journal.pntd.0001465)

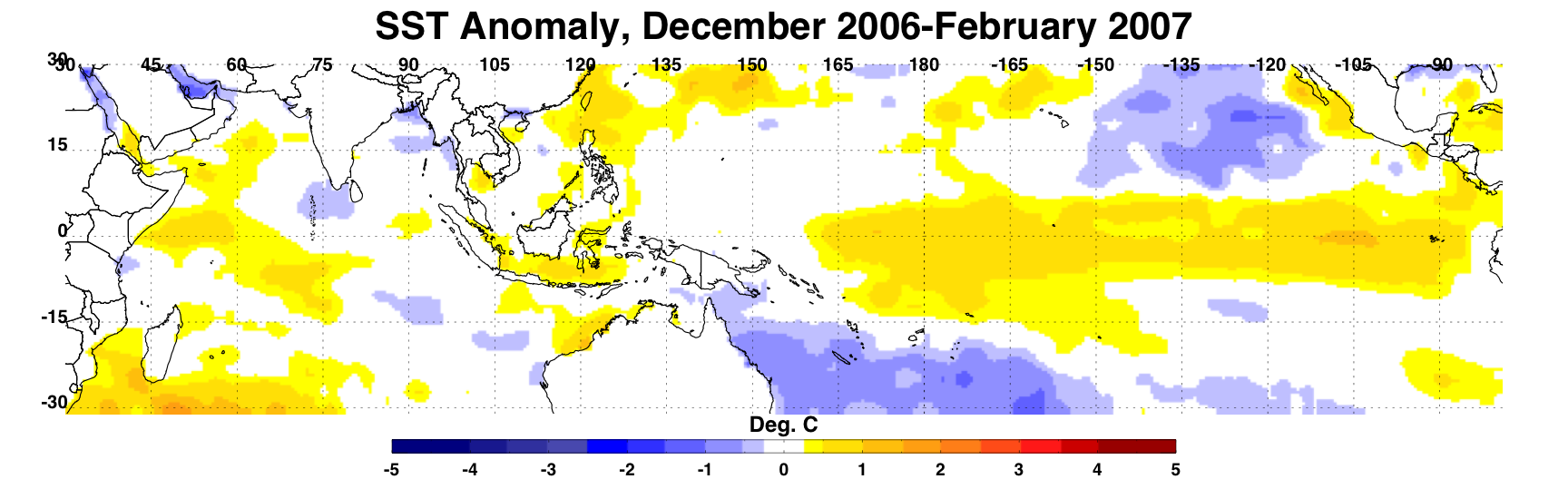

Supplement: Figure S1 — SST anomalies during the peak of the El Niño event from December, 2006–February, 2007. Global seasonal equatorial sea surface temperature anomalies during the peak of the El Niño event December, 2006–January, 2007. (TIFF) [file pntd.0001465.s002.tif]

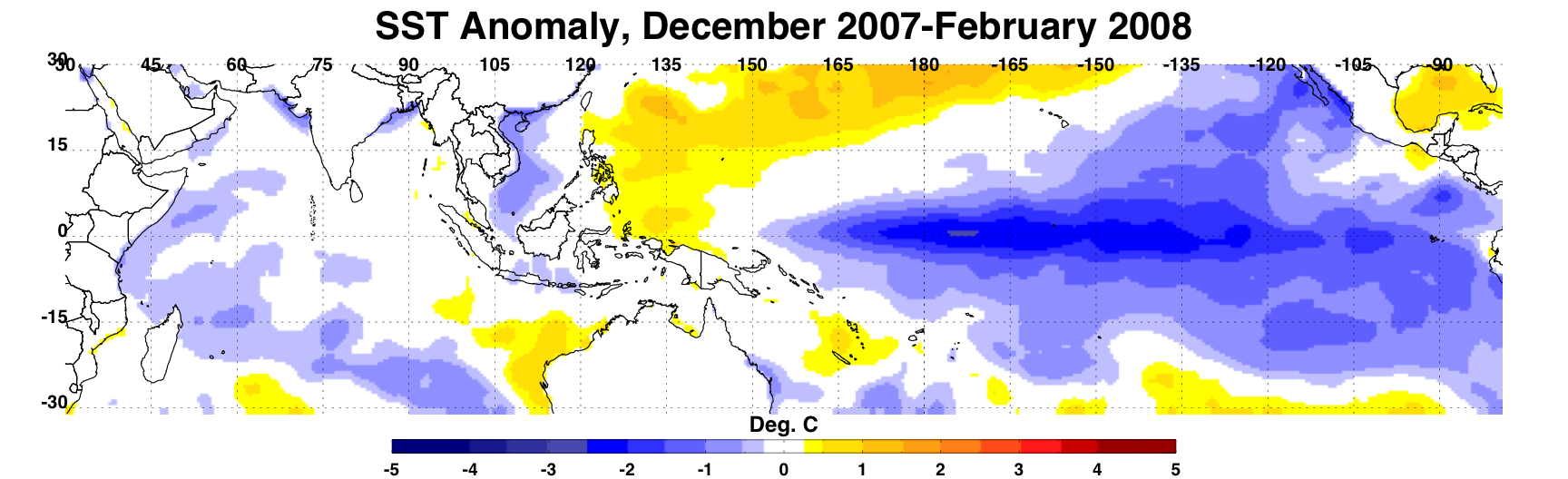

Supplement: Figure S2 — SST anomalies during the peak of the La Niña event from December, 2007–February, 2008. Global seasonal equatorial sea surface temperature anomalies during the peak of the La Niña event December, 2007–February, 2008. (TIFF) [file pntd.0001465.s003.tif]

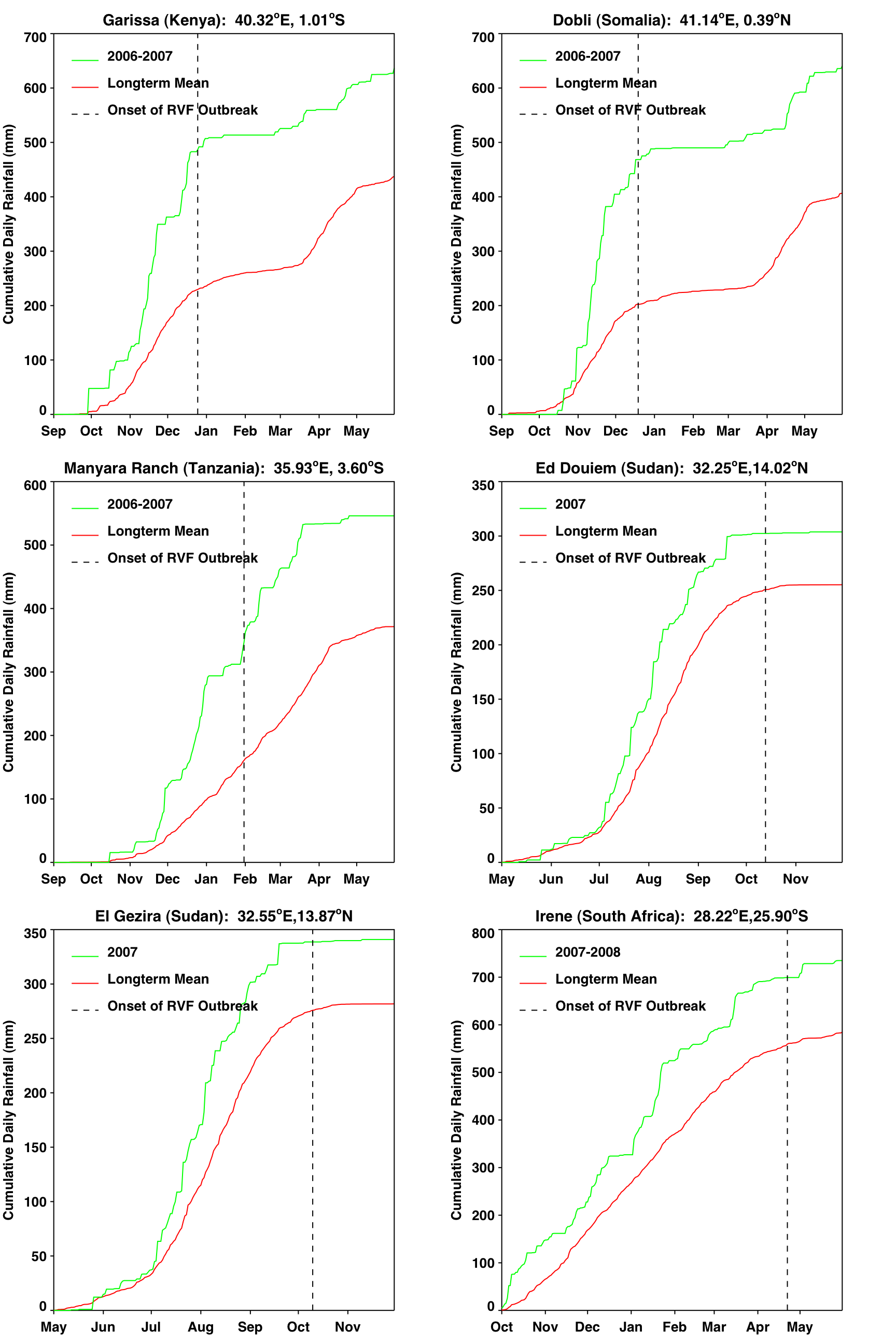

Supplement: Figure S3 — Cumulative daily rainfall profiles for periods of Rift Valley fever activity for selected outbreak sites. Cumulative daily rainfall (green lines) profiles for periods of Rift Valley fever activity and mean long-term cumulative daily rainfall (red lines) for sites with reported Rift Valley fever activity. Dotted line represents when the first case of Rift Valley fever was identified at each location. Each of the outbreak locations was preceded by above-normal rainfall for 3–4 months. (TIFF) [file pntd.0001465.s004.tif]

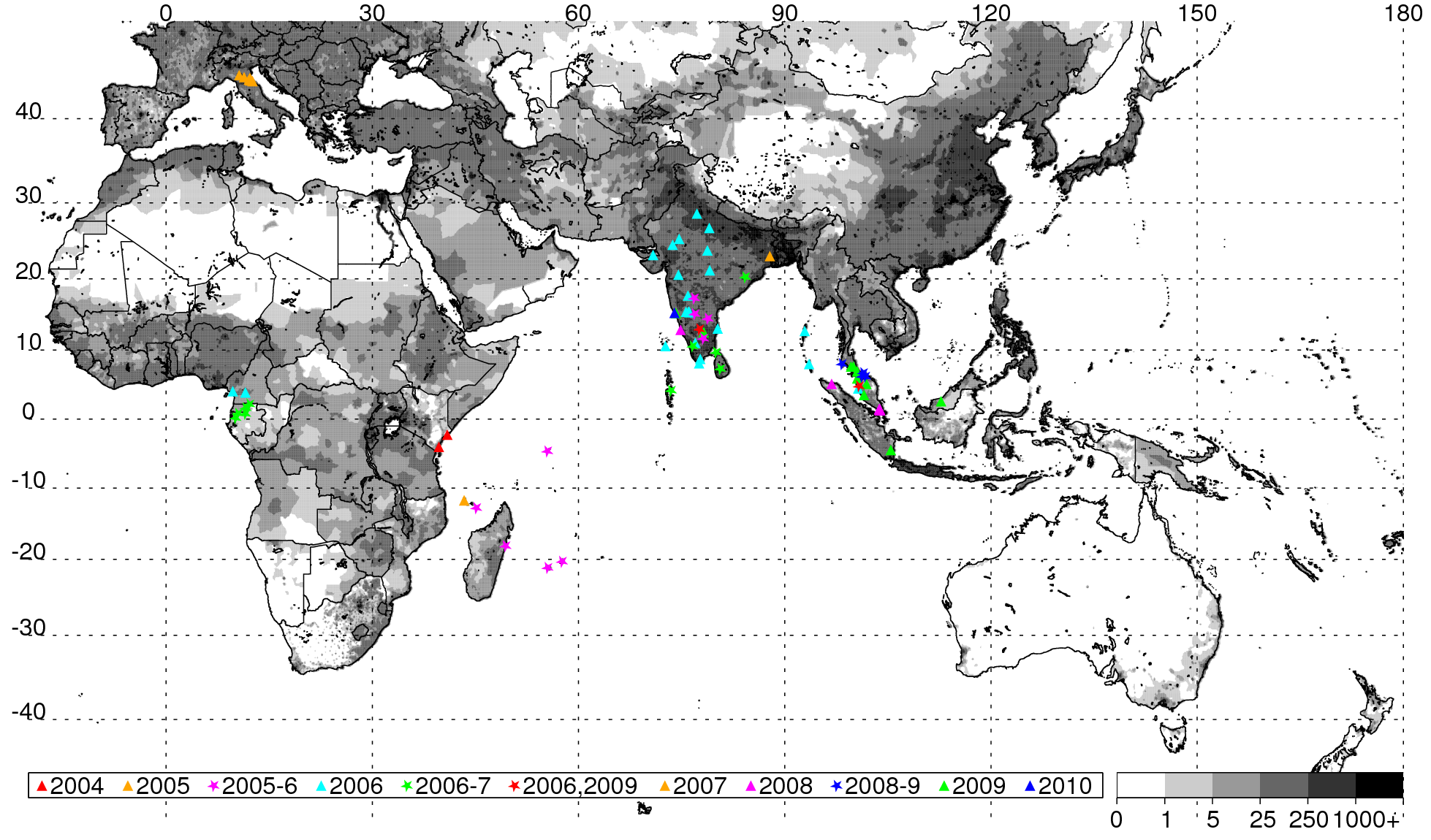

Supplement: Figure S4 — Distribution of 2004–2010 chikungunya outbreaks in relation to human population density. Each symbol represents the year(s) when an outbreak was reported at a specific geographic location. Most chikungunya activity has occurred in locations with high population densities (>500 people per square kilometre). (TIF) [file pntd.0001465.s005.tif]
